# Comprehensive genomic features indicative for Notch responsiveness

**DOI:** 10.1093/nar/gkae292

**Published:** 2024-04-22

**Authors:** Benedetto Daniele Giaimo, Tobias Friedrich, Francesca Ferrante, Marek Bartkuhn, Tilman Borggrefe

**Affiliations:** Institute of Biochemistry, Justus-Liebig-University Giessen, Friedrichstrasse 24, 35392 Giessen, Germany; Institute of Biochemistry, Justus-Liebig-University Giessen, Friedrichstrasse 24, 35392 Giessen, Germany; Biomedical Informatics and Systems Medicine, Justus-Liebig-University Giessen, Aulweg 128, 35392 Giessen, Germany; Institute of Biochemistry, Justus-Liebig-University Giessen, Friedrichstrasse 24, 35392 Giessen, Germany; Biomedical Informatics and Systems Medicine, Justus-Liebig-University Giessen, Aulweg 128, 35392 Giessen, Germany; Institute for Lung Health, Aulweg 132, 35392 Giessen, Germany; Institute of Biochemistry, Justus-Liebig-University Giessen, Friedrichstrasse 24, 35392 Giessen, Germany

## Abstract

Transcription factor RBPJ is the central component in Notch signal transduction and directly forms a coactivator complex together with the Notch intracellular domain (NICD). While RBPJ protein levels remain constant in most tissues, dynamic expression of Notch target genes varies depending on the given cell-type and the Notch activity state. To elucidate dynamic RBPJ binding genome-wide, we investigated RBPJ occupancy by ChIP-Seq. Surprisingly, only a small set of the total RBPJ sites show a dynamic binding behavior in response to Notch signaling. Compared to static RBPJ sites, dynamic sites differ in regard to their chromatin state, binding strength and enhancer positioning. Dynamic RBPJ sites are predominantly located distal to transcriptional start sites (TSSs), while most static sites are found in promoter-proximal regions. Importantly, gene responsiveness is preferentially associated with dynamic RBPJ binding sites and this static and dynamic binding behavior is repeatedly observed across different cell types and species. Based on the above findings we used a machine-learning algorithm to predict Notch responsiveness with high confidence in different cellular contexts. Our results strongly support the notion that the combination of binding strength and enhancer positioning are indicative of Notch responsiveness.

## Graphical Abstract

**Graphical Abstract:**
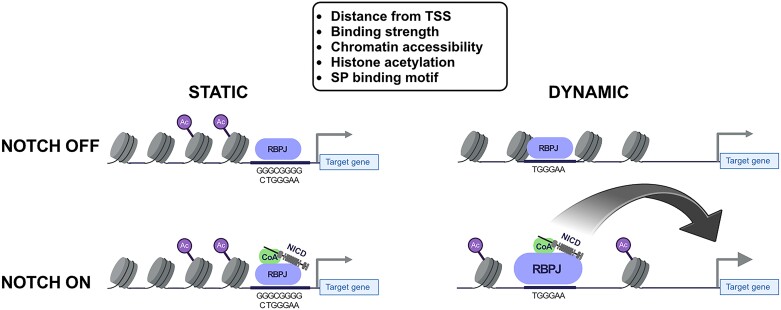

## Introduction

Signal transduction pathways enable transmission of extracellular signals, environmental changes or mechano-transduction into changes in gene expression. For several signaling pathways, it is known that ligand or stimulus-dependent changes lead to the translocation into the nucleus of a pathway-specific transcription factor (TF; i.e. SMADs and NFκB). Alternatively, TF levels in the nucleus may remain constant but changes in the associated cofactors occur (i.e. β-catenin/TCF and RBPJ). While changes in gene expression have been already studied using transcriptomics approaches, studies combining TFs occupancy combined with dynamic gene expression remain scarce.

In regard to the Notch signaling pathway, ligand binding leads to the release of the Notch intracellular domain (NICD) resulting in the assembly of a coactivator complex, also known as Notch transcriptional complex (NTC) (1,2). This complex consists of TF RBPJ, also known as CSL (*Homo sapiens* CBF1, *Drosophila melanogaster* Suppressor of Hairless, and *Caenorhabditis elegans* Lag-1), together with NICD and, among others, the acetyltransferase EP300 to promote the expression of Notch target genes. In absence of Notch signaling, RBPJ and HDAC-containing associated corepressors inhibit Notch target genes expression. Several studies have focused on the identification and characterization of components of both the corepressor and coactivator complexes, thereby elucidating on the regulation of the chromatin environment at Notch target genes (3–13). The exact mechanism of repression is well-documented and includes additional cofactors such as the SHARP/NCoR complex recruiting HDACs (8,9,12) and histone demethylases (6,10,14). Several studies have also analyzed the genome-wide distribution of RBPJ in various models (15–25). Although it was previously thought that RBPJ binds to its cognate enhancers regardless of Notch status, more recent studies have revealed a different scenario. In fact, Notch activation significantly increases the binding of RBPJ (15,21,24,26). So far, the functional meaning and the underlying molecular mechanism(s) determining these changes in RBPJ occupancy remained unclear.

Here, we determined either ‘static’ or ‘dynamic’ RBPJ sites in a mouse progenitor T-cell line with characteristic constitutive Notch signal activity. Only dynamic occupancy of RBPJ correlates with Notch-dependent changes in the chromatin state and gene expression. Dynamic RBPJ occupancy characterizes Notch responsiveness also in human T-ALL cell lines as well as breast cancer cell lines. Leveraging a random forest model, we can identify Notch responsiveness using a machine-learning algorithm solely based on genome-wide occupancy of RBPJ in a variety of cell types.

## Materials and methods

### Cell culture and treatments

Mouse leukemia preT-cells (Beko) were grown at 37°C under 5% CO_2_ in Iscove's modified Dulbecco medium (IMDM, Gibco 21980-065) supplemented with 2% fetal bovine serum (Pan Biotech), 5 mg/l insulin (Sigma-Aldrich), 0.3 mg/ml Primatone, nonessential amino acids (Gibco) and penicillin/streptomycin (Gibco).

*Drosophila melanogaster* Schneider cells were grown in Schneider's *Drosophila* medium (Gibco 21720024) supplemented with 10% fetal bovine serum (Gibco 10270-106), Glutamine (Gibco 25030-024) and penicillin/streptomycin (Gibco).

Beko cells were treated with 10 μg/ml GSI (DAPT; Alexis ALX-270-416-M025), 0.01 μg*/*ml apicidin (Sigma-Aldrich A8851) or with DMSO as control for 24 h. In the case of the washout experiments, Beko cells were incubated with 10 μg/ml GSI (supplemented freshly at 24 h after washing away the old one) for 48 h. Subsequently, cells were collected, washed and placed in culture for additional 24 h before performing the experiment.

### Protein extracts and western blotting

Nuclear extracts were prepared by washing the cells twice in PBS and resuspending them in Hypotonic buffer (20 mM Hepes pH 7.9, 20 mM NaCl, 5 mM MgCl_2_, 10% glycerol, 0.2 mM PMSF). After 20 min incubation on ice, samples were centrifuged (4000 rpm, 10 min, 4°C) and the nuclei were washed twice with ice-cold PBS. Nuclei were lysed in Hypertonic Buffer (20 mM Hepes pH 7.9, 1 mM MgCl_2_, 300 mM NaCl, 0.2% NP-40, 25% glycerol, 0.2 mM PMSF, 1× Protease inhibitor mix, 0.3 mM DTT) and incubated 20 min on ice. After centrifugation (14 000 rpm, 5 min, 4°C), the supernatants were collected and protein concentration was measured by Bradford assay (Sigma-Aldrich). Extracts were boiled in presence of SDS loading buffer and analyzed by western blotting.

For western blotting purposes, proteins were resolved in SDS polyacrylamide gels and transferred to a Nitrocellulose membrane (Amersham) by wet blotting.

Membranes were blocked in 5% milk/TBST (1× TBS, 0.1% Tween 20) and incubated over night with the desired antibody diluted in 5% milk/TBST [1:1000 H3 (abcam ab1791); 1:1000 Val1744 cleaved NICD1 (Cell Signaling Technology 4147)]. Membranes were washed in TBST and incubated 1 h at room temperature with secondary antibody diluted 1:5000 in 5% milk/TBST [anti-rabbit IgG HRP (Cell Signaling 7074S)]. Membranes were washed in TBST and incubated at room temperature with ECL solution. Chemiluminescence was detected with a Vilber Fusion FX7 system.

### RNA extraction, libraries and sequencing

Total RNA was purified using the RNeasy Mini Kit (Qiagen #74104), the QIAshredder (Qiagen #79654) and the DNase I (Qiagen #79254) accordingly to manufacturer′s instructions. Libraries were prepared using the TruSeq® Stranded Total RNA LT—Ribo-Zero Gold kit (Illumina RS-122-2301/2) and sequenced on a NextSeq device.

### RNA-seq and microarray analysis

Basic summary statistics for the RNA-Seq experiments are provided in Supplementary Table S11.

Previously published microarray and RNA-Seq datasets used in this study are indicated in Supplementary Table S11. Microarray data was downloaded within R v. 4.0.3 with the *getGEO* function of the GEOquery (Davis and Meltzer 2007) package. Since the data were already log normalized, the log_2_FC was calculated by subtracting the treatment values from the control value

RNA-Seq analysis was performed within R using a custom-made version of the systemPipeR (27) R/BioConductor package. Raw sequencing reads were adaptor and quality trimmed using trimGalore v. 0.6.5 (https://github.com/FelixKrueger/TrimGalore. The quality of the trimming was validated by visual inspection after using systemPipeR’s *seeFastq* function. Trimmed FASTQ files were aligned against the mouse (mm9) or human (hg19) genome using HISAT v. 2.2.1 (28) with the parameters ‘–phred33 –k 1 –min-intronlen 30 –max-intronlen 3000’ and stored as sequence alignment maps. Conversion from sequence alignment map format to binary alignment map format (BAM) was done using the *runCommandline* function of the systemPipeR package. These BAM files were quality checked by using systemPipeR’s *alignStats* function and subsequently a gene reads table was calculated by the *summarzieOverlaps* function of the GenomicAlignments (29) R/BioConductor package and the corresponding Gene Transfer Format (GTF) file (Illumina's iGenomes). Normalization and detection of deregulated genes was performed using the DESeq2 v. 1.28.1 package (30). Criteria for the definition of significantly deregulated genes were log_2_FC >1 or <–1 and adjusted *P*-value <0.05.

### Chromatin immunoprecipitation (ChIP), libraries and sequencing

ChIP experiments were performed as previously described (25). Briefly, cells were fixed in 1% FMA for 30 min at room temperature. The FMA reaction was blocked for 5 min at room temperature by adding 1/8 volume of 1 M glycine pH 7.5. Cells were washed twice with PBS and resuspended in 1 ml of SDS Lysis Buffer (1% SDS, 10 mM EDTA, 50 mM Tris–HCl pH 8.1). After incubation on ice for 10 min, samples were sonicated using a Covaris System S220 AFA (28 cycles, 30 s ON, 30 s OFF). The chromatin was diluted in ChIP Dilution Buffer (0.01% SDS, 1.1% Triton X-100, 1.2 mM EDTA, 16.7 mM Tris–HCl pH 8.1, 167 mM NaCl) and pre-cleared with protein-A-Sepharose beads (GE Healthcare 17-5280-02) for 30 min at 4°C. The chromatin was subsequently incubated over night with the proper amount of the desired antibody and the antibodies were immobilized with 40 μl protein-A-Sepharose beads for 1 h at 4°C with rotation. Depending on the antibody, different combinations of the following washing buffers were used: low salt washing buffer (0.1% SDS, 1% Triton X-100, 2 mM EDTA, 20 mM Tris–HCl pH 8.1, 150 mM NaCl), high salt washing buffer (0.1% SDS, 1% Triton X-100, 2 mM EDTA, 20 mM Tris–HCl pH 8.1, 500 mM NaCl), LiCl washing buffer (1% NP-40, 1 mM EDTA, 10 mM Tris–HCl pH 8.1, 0.25 M LiCl) and TE buffer (10 mM Tris–HCl pH 8.0, 1 mM EDTA). Chromatin was eluted from beads with Elution Buffer (50 mM Tris–HCl pH 8.0, 10 mM EDTA pH 8.0, 1% SDS) and crosslinks were reverted at 65°C over night. After diluting the SDS with TE buffer, samples were incubated with RNase A (Thermo Scientific EN0531) at 37°C for 2 h and subsequently with Proteinase K (Invitrogen 25530-049) at 55°C for 2 h. After extraction with phenol*/*chloroform/isoamylic alcohol, the DNA was purified using the Qiaquick PCR cleanup kit (Qiagen 28104).

The following antibodies were used: H3K4me1 (abcam ab8895), H3K4me3 (Diagenode pAb-003-050), H3K9ac (abcam ab4441), H3K9me1 (Cell Signaling 14186), H3K9me2 (Active Motif 39753), H3K9me3 (Diagenode C15410193), H3K18ac (Cell Signaling #9675S), H3K27me1 (Diagenode pAb-045–050), H3K27me2 (Diagenode pAb-046-050), H3K27me3 (Active Motif 39155), H3K36me1 (Cell Signaling #14111), H3K36me2 (abcam 176921), H3K36me3 (Cell Signaling #4909S), or RBPJ (Cell Signaling Technology 5313).

ChIP experiments were analyzed on a StepOnePlus™ sequence detector system (Applied Biosystem) using specific oligonucleotides and double-dye probes (Supplementary Table S12).

In the case of the ChIP-Seq, chromatin from *D. melanogaster* Schneider cells was used for spike-in purposes (each 25 μg of mouse chromatin, 50 ng or 25 ng of *Drosophila* chromatin were used in ChIP versus histone proteins or TFs respectively). 2 μg of anti-His2Av (Active Motif 61686) were added to each immunoprecipitation for spike-in purposes. Libraries were prepared using the Diagenode MicroPlex Library Preparation kit v2 (Diagenode C05010012) or the Diagenode MicroPlex Library Preparation kit v3 (Diagenode C05010001) following manufacturer's instructions with few modifications. Libraries were purified with Agencourt AMPure XP Beads (Beckman Coulter, #A63881), quantified, analyzed on a Tapestaion device (Agilent) and pooled. Finally, sequencing was performed on a HS2500 or a NovaSeq device.

### ChIP-seq analysis

Basic summary statistics for the ChIP-Seq experiments are provided in Supplementary Table S11.

The previously published ChIP-Seq datasets used in this study are indicated in Supplementary Table S11. Primary processing of raw sequencing reads until generation of BAM files was done as described for the RNA-Seq analysis. HISAT2 was used for both single- and paired end reads with ChIP-Seq specific parameters ‘-k 1 –no-spliced-alignment –phred33’. Duplicated reads were removed from BAM files using Picard Tools (available at http://broadinstitute.github.io/picard/) MarkDuplicates function with additional parameters ‘REMOVE_SEQUENCING_DUPLICATS = true REMOVE_DUPLICATES = true’. MACS2 v. 2.2.7.1 (31) was used with a q-value threshold of 0.01 to call peaks for the individual BAM files with or without input. When ChIP-Seq replicates were available, MSPC v.4.0.0 (32) was used with parameters ‘-r bio -w 1e-6 -s 1e-10’ to validate the called peaks and determine the set of ‘true positive peaks’. Only if a true positive RBPJ peak was conserved in three out of five control replicates (Beko) or 2 out of 2 ‘Washout’ replicates (MB157, HCC1599, CUTLL1, IC8) it was selected as a real binding site. For ChIP-Seq analysis of histone modifications (H3K27ac, H3K4me1, H3K4me3, H3K18ac, H3K9ac) a peak had to be conserved in three out of four (DMSO/GSI or GSI/Washout) replicates. Peaks were filtered for ENCODE blacklisted regions (downloaded from https://sites.google.com/site/anshulkundaje/projects/blacklists). Read counts per site were collected using the *summarzieOverlaps* function of the GenomicAlignments R/BioConductor package. DESeq2 was used to calculate normalization factors per replicate based on these read counts. DeepTools (33) *bamCoverage* function was used to calculate normalized BigWig files using the normalization factors provided by DESeq2 or the RPKM normalization for the remaining files. The BigWig files were used to visually inspect the quality of the ChIP-Seq experiments and the effectiveness of the peak calling in IGV (34).

DESeq2 was used for the identification of dynamic RBPJ binding sites and deregulation of ATAC-Seq and histone marks. In case of ATAC-Seq or histone modifications the window for detection of changes was from 500 bp upstream to 500 bp downstream of the RBPJ sites. All RBPJ binding sites with a log_2_FC <–0.5 upon GSI (or >0.5 upon washout of GSI) were selected as dynamic sites. DeepTools *computeMatrix* function was used to calculate a score per genome region matrix based on the normalized BigWig files and the binding sites as a reference. These matrices were used to plot the heat maps using deepTools *plotHeatmap* function or the average binding signal as line plots within R. For the identification of binding motifs at RBPJ sites the MEME-Suite v. 5.3.3 (35) was used. For the identification of head to head RBPJ binding motifs the *vmatchPattern* (36) function was used with the allowance of up to two mismatches. Position of the RBPJ binding sites in relation to the TSS was calculated and plotted using the ChIPseeker (37) package. Known mouse mm9 CpG islands were downloaded from UCSC table browser. Snapshots were generated using Gviz (38).

Identification of chromatin states was performed using ChromHMM (39). For this purpose, the BAM files from histone modifications ChIP-Seq and ATAC-Seq were converted into binarized data with a bin size of 500bp using the *BinarizeBam* function. Based on the binarized data, a 25 state model was generated using the *LearnModel* function. State 4, which represents the absence of any signals, has been removed from the final figures.

Annotation of RBPJ binding sites to their corresponding gene was performed using an in-house tool (that works in a comparable manner to basal plus extension of GREAT) in combination with the corresponding GTF file. Genes that are associated with both a dynamic and a static binding site were assigned as dynamic. The gene annotations were used for the gene over-representation analysis (GO & KEGG database) calculated with the clusterProfiler (40) package and plotted with ggplot2 (41). Additionally, the GREAT (42,43) web interface was used to identify the enrichment of GO terms or the Mouse Phenotype Single KO ontologies based on static or dynamic RBPJ binding sites.

### Prediction model

In order to predict dynamic and static RBPJ binding sites we used MSPC’s *P*-value (as a proxy for the quality of binding regions), the position feature as defined by the ChIPseeker *annotatePeaks* function and the occurrence of the SP1 or RBPJ binding motif. In detail, the model was trained using five features: First, the normalized *P*-values were calculated by MSPC (32) for each peak, which is based on the combination of the individual *P*-values of the RBPJ ChIP-Seq replicates generated by the peak caller (PeakRanger). MSPC *P*-values were min-max normalized to make it comparable between different data sets, representing the lowest p-value for the most significant peak and the highest p-value for the least significant one. Second, the genomic annotation of RBPJ peaks identified by ChIPseeker was used. ChIPseeker identifies the specified features (promoter, intron, exon, intergenic, downstream, 5′UTR or 3′UTR) of each binding location. The features were extracted from the corresponding GTF file, which is used to store transcript annotations. After testing different features, intron and exon regions were combined to represent gene bodies. Lastly, the occurrence of SP1 or RBPJ binding motifs was used as a feature for the prediction. To do so, the FIMO tools of the MEME suite were used to identify peaks carrying the SP1 motif, the canonical RBPJ (TGGGAA) or the degenerate RBPJ motif (TGRGAA) found predominantly at dynamic sites.

The model was established using a combination of the Beko and MB157 datasets. To generate a final test set for the model, the *createDataPartition* function of the caret (44) package was used to split the dataset into 85% model data and 15% test data. The 85% model set was further divided into 80% training and 20% validation sets. The random forest model was implemented based on the training data using the *randomForest* function of the randomForest (45) package. One random model with a mean correct prediction (true positive) of dynamic sites over 65% in the validation set and the external IC8 set was chosen as the final one.

This model was tested and validated on the HCC1599 and CUTLL1 cells. Since the location proximal and distal to the TSS are key features for static or dynamic RBPJ sites, it was important to test whether the model actually predicts dynamic and static locations or simply divides them into proximal and distal regions. To test this, the falsely predicted static and dynamic sites were compared for their associated genomic features. Indeed, both groups where most frequently associated with intergenic sites, indicating that the model does not simply separate into promoter or intergenic binding sites. The accuracy was calculated as the ratio between correctly predicted locations and all predictions (Correct predicted/all predictions). Area under the ROC curve was calculated using the ROCR package (https://doi.org/10.1093/bioinformatics/bti623) and plotted using ggplot2.

### Clusters of enhancers

The BAM files of the two replicates for H3K27ac ChIP-Seq in Beko control cells were merged using samtools’ *merge* function. This merged BAM was used to call peaks using MACS2 (31) with the –nomodel –mm9’. The called peaks together with the merged BAM file was used as an input for the identification and ranking of enhancer clusters using ROSE (46).

### ATAC-Seq

ATAC-Seq was performed with the ATAC-Seq kit (Active Motif 53150) accordingly to manufacturer′s instructions and samples were sequenced on a NovaSeq device.

### ATAC-Seq analysis

Basic summary statistics for the ATAC-Seq experiments are provided in Supplementary Table S11. ATAC-Seq primary analysis was performed identical to ChIP-Seq. MACS2 without input was used to for peak calling. Only peaks that were conserved in three out of six replicates were selected as real ATAC-Seq sites after removing blacklisted regions.

### Software

Statistical analysis of ChIP-qPCR data was done using GraphPad Prism. Graphical abstract was created with BioRender.com. All figures were assembled with Affinity Designer.

## Results

### Identification of dynamic binding of transcription factor RBPJ

**Figure 1.**
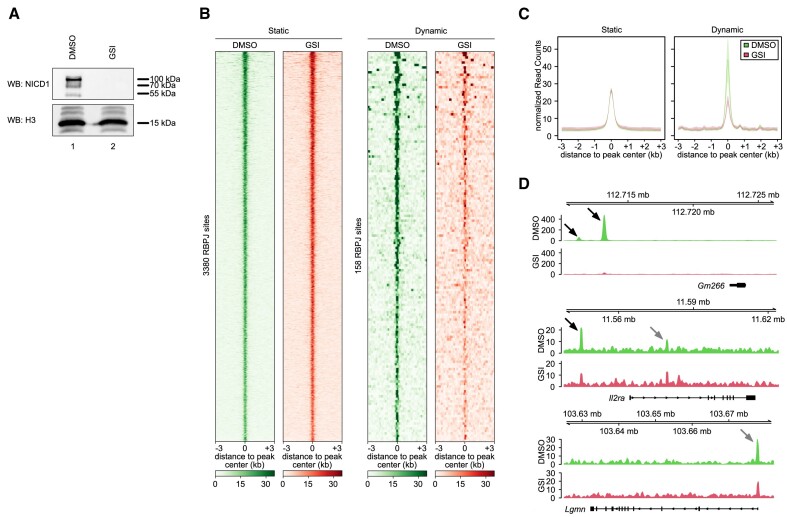
Identification of static and dynamic RBPJ binding sites in Beko cells. (**A**) The active cleaved NICD1 protein disappears after GSI treatment for 24 h. Beko cells were treated for 24 h with 10 μg/ml GSI or DMSO as control and the nuclear extracts (NE) were analyzed by Western blotting versus the endogenous cleaved Notch1 protein (NICD1) or H3 as loading control. (**B**) Heat map showing the static and dynamic RBPJ binding sites identified in control Beko cells and their behavior upon treatment with GSI. Beko cells were treated for 24 h with 10 μg/ml GSI or DMSO as control and the genome-wide binding of RBPJ was investigated by ChIP-Seq. (**C**) Line plots showing the average RBPJ binding signal for static and dynamic sites. Outline depicting the standard deviation of the replicates. Beko cells were treated for 24 h with 10 μg/ml GSI or DMSO as control and the genome-wide binding of RBPJ was investigated by ChIP-Seq. (**D**) Representative snapshots showing dynamic RBPJ binding at *Gm266* and static RBPJ binding at *Lgmn*. *Il2ra* is characterized by both static and dynamic RBPJ binding sites. Black arrows indicate dynamic binding sites while gray arrows indicate static RBPJ binding sites.

In order to study the genome-wide occupancy of TF RBPJ, we performed ChIP-Seq analysis in a mouse progenitor T-cell line called Beko (6,16). Beko cells are constitutively active for Notch signaling and formation of NICD1 can be blocked by treatment with γ-secretase inhibitor (GSI; Figure 1A). In control DMSO-treated Beko cells, a total of 3538 RBPJ binding sites were detected (Supplementary Table S1). This number of binding sites is comparable to previously published data in mouse mature T-cells (47). When we compared the RBPJ binding profile in presence or absence of GSI we observed that the majority of sites (3380) remained unaffected by GSI treatment, but a small fraction (158 sites) showed a marked reduction of RBPJ binding (log_2_ fold change cut-off < –0.5) upon addition of GSI; We refer to these sites as either static or dynamic sites, respectively (Figure 1B,C and Supplementary Table S1). Interestingly, RBPJ binding is stronger at dynamic sites compared to static sites when the Notch pathway is active (Figure 1C). Differences in binding strength could be validated by ChIP-qPCR (Supplementary Figure S1A) and representative examples of dynamic and static RBPJ binding sites are shown for *Gm266*, *Il2ra*, *Lgmn*, *Dennd2d*, *Sufu*, *Hey1*, *Hes1*, *Notch3* and *Dtx1* (Figures 1D and S1B). The canonical RBPJ motif ‘TGGGAA’ was identified (Supplementary Figure S1C and Supplementary Table S2): The RBPJ motif identified at dynamic sites had a less conserved ‘G’, but both purines occurred with equal frequency (TGRGAA). The SP (Specificity Protein family) motifs are preferentially identified at static sites, whereas the RBPJ motif marks a large fraction of the dynamic sites (Supplementary Figure S1C and Supplementary Table S2). In addition, static sites are enriched for NRF1, NFYA/NFYB/DUX, FOXJ3/ZF384 and ZBTB7A/GABPA/ELK4 motifs while dynamic sites are enriched for the TCF3/TCF4 motif (Supplementary Table S2). Previous studies have described that Notch target genes are regulated by dimeric RBPJ complexes that bind to dimeric DNA binding motifs oriented head-to-head and with a distance of 15–17 nucleotides (1,48–51). Interestingly, we observed that dimeric RBPJ binding sites are preferentially enriched within the group of dynamic sites (Supplementary Figure S1D, E).

We have previously shown that HDAC3 inhibition or hypoxia treatment in Beko cells reduce the NICD1 stability leading to downregulation of the Notch response (16,52). We reasoned that reduced NICD1 stability could lead to reduced RBPJ occupancy at dynamic but not static sites so we performed RBPJ ChIP-Seq in Beko cells treated with apicidin and we reanalyzed our previously published RBPJ ChIP-Seq upon hypoxia induction (52): We observed that both apicidin treatment and hypoxia induction result in reduced occupancy of RBPJ at dynamic but not at static sites (Supplementary Figures S2 and S3) validating the authenticity of static and dynamic sites.

Taken together, our results in Beko cells show that RBPJ occupancy is strongly reduced at only a small fraction of binding sites upon pharmacological inhibition of the Notch pathway.

### Characterization of dynamic RBPJ binding sites

**Figure 2.**
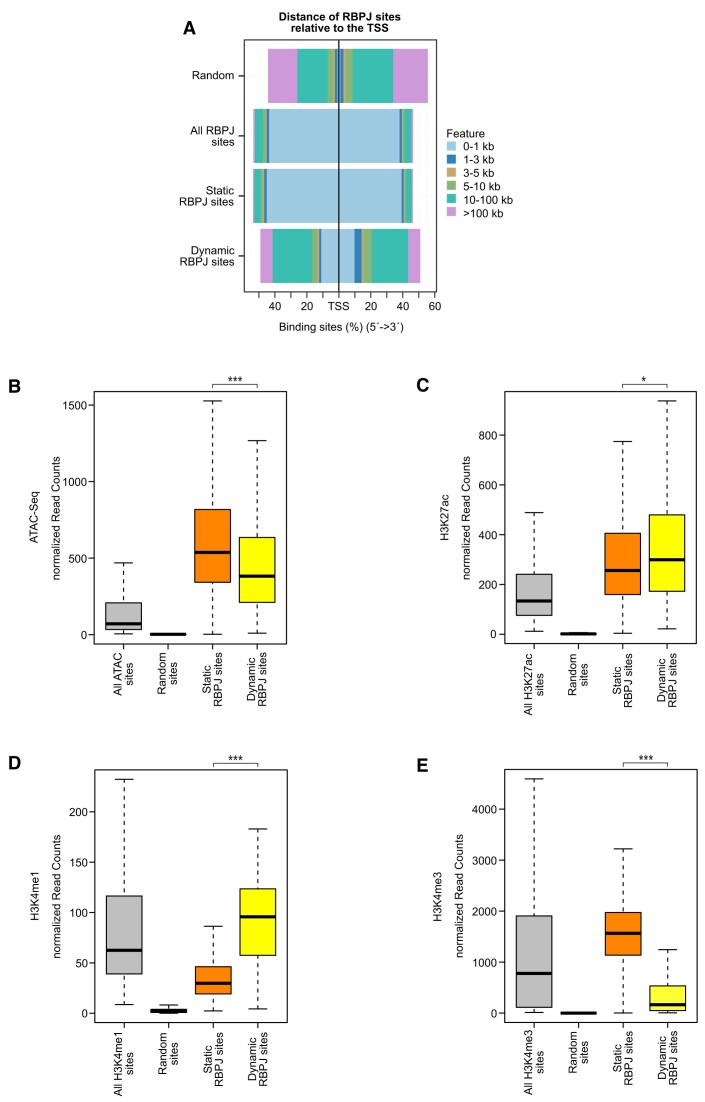
Dynamic RBPJ sites represent distal enhancers. (**A**) Distance of all, static and dynamic RBPJ sites to the next transcription starting site (TSS). The figure includes the genomic background distribution (random). (B–E) Beko cells were analyzed by ATAC-Seq and ChIP-Seq to characterize the chromatin landscape at static and dynamic RBPJ sites or at random sites and all detected sites of the given mark (or ATAC-seq) as control. (**B**) Box plot showing that both dynamic and static sites display an open chromatin configuration as measured by ATAC-Seq. The chromatin accessibility is observed at both static and dynamic RBPJ sites but slightly higher at static compared to dynamic ones. (**C**) Box plot showing that H3K27ac is higher at dynamic compared to static RBPJ sites. (**D**) Box plot showing that H3K4me1, a typical enhancer mark, is higher at dynamic compared to static RBPJ sites. (**E**) Box plot showing that H3K4me3 is higher at static compared to dynamic RBPJ sites. Wilcoxon rank sum tests (***P <0.001, *P <0.05).

Next, we characterized the differences between static and dynamic RBPJ binding sites, taking genomic and chromatin features into account. Most static sites are located close to transcriptional start sites (TSSs; Figures 2A and S4A). In contrast, dynamic sites were preferentially found in intra- and intergenic regions (Figures 2A and S4A). Both static and dynamic sites were associated with open chromatin regions as measured by ATAC-Seq (Figure 2B). In addition, both groups of RBPJ sites were enriched for active chromatin marks H3K27ac, H3K18ac and H3K9ac (Figures 2C, S4B, C). In line with the differences in binding position (Figure 2A), enhancer mark H3K4me1 is highly enriched at dynamic sites whereas H3K4me3, predominantly found at TSSs, was highly enriched at static sites (Figure 2D, E, respectively). Of note, the higher enrichment of H3K4me3 and H3K9ac at static sites compared to the dynamic ones (Figures 2E and S4C) reflects their co-localization at TSSs that has been previously described (53,54), further supporting the notion that static RBPJ sites are preferentially enriched at promoter-proximal enhancers. Analysis of the chromatin states further confirmed that dynamic RBPJ sites represent distal enhancers while static sites localize to proximal enhancers (Supplementary Figures S4D, E). In addition, RBPJ binding highly overlaps with CpG islands (CGI; Supplementary Figure S5A) and closer inspection unveiled that static RBPJ sites are more associated with CGI than dynamic sites (Supplementary Figure S5B), in line with the notion that promoters are CGI-rich (55,56). As already stated above, the higher enrichment of H3K4me3 at static sites further reflects their co-localization at promoters, which has been previously described (53). We have previously described the role of the lysine methyltransferase 2D (KMT2D) in the dynamic regulation of H3K4me3 in Notch target gene activation (9). Therefore, we focused on this mark to have a detailed genome-wide view of its regulation in response to Notch signaling. We observed that static H3K4me3 sites that overlap with RBPJ sites are closer to the TSS compared to dynamic H3K4me3 sites that overlap with RBPJ (Supplementary Figure S5C). In addition, co-localization of static H3K4me3 sites and RBPJ sites overlap more frequently with CGIs (Supplementary Figure S5D). These data suggest that H3K4me3 is more dynamic at distal than promoter proximal RBPJ binding sites, in line with our previous observations (9).

In conclusion, dynamic and static RBPJ binding sites reveal both high levels of chromatin accessibility and activity but differ in regard to distance to the TSS, which is reflected in chromatin marks.

### Transcriptional response to Notch activation is preferentially associated with dynamic sites

**Figure 3.**
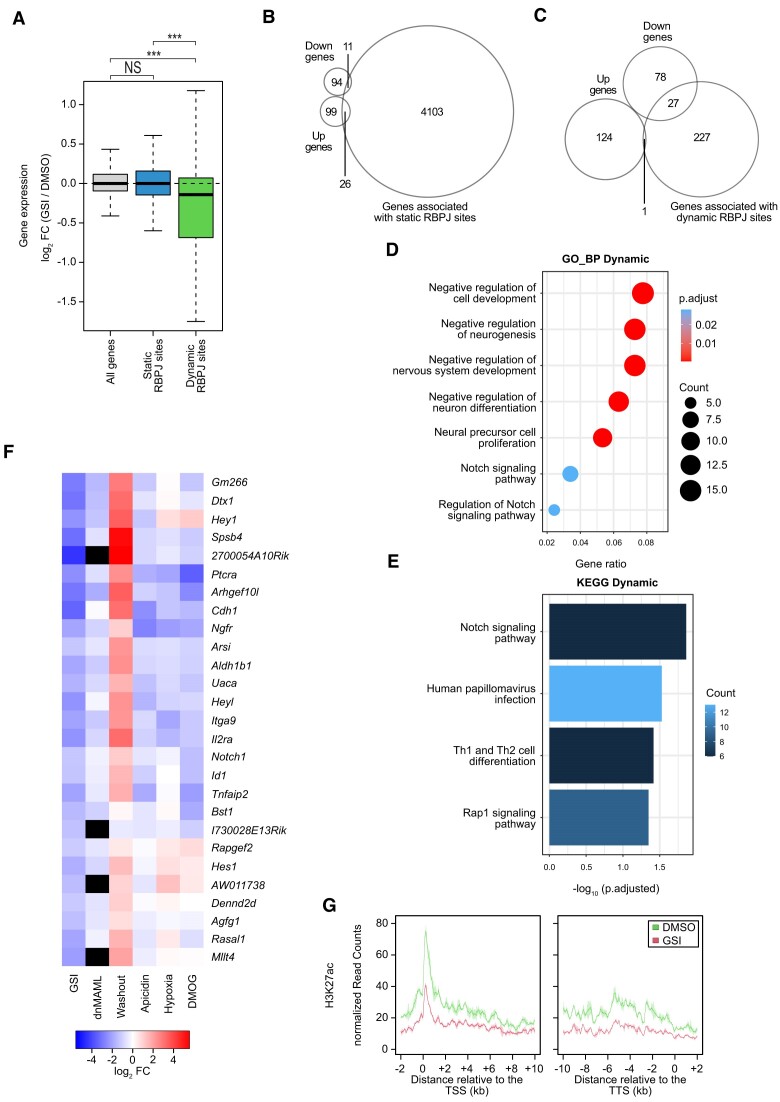
Transcriptional Notch responsiveness is associated with dynamic RBPJ sites. Beko cells were treated for 24 h with 10 μg/ml GSI or DMSO as control. (**A**) Box plot showing the effects of GSI treatment on the expression of genes associated with static or dynamic RBPJ sites. Wilcoxon rank sum tests (***P <0.001, NS = not significant). (**B**) Venn diagram showing the overlap between genes associated with static RBPJ sites and genes upregulated (up) or downregulated (down) upon GSI treatment. (**C**) Venn diagram showing the overlap between genes associated with dynamic RBPJ sites and genes upregulated (up) or downregulated (down) upon GSI treatment. (**D**) ORA based on the gene ontology (GO) database for the group of dynamic RBPJ sites identified in Beko cells. The panel depicts the results for the ‘Biological Process’ (BP) class. Full list of the GO terms identified in this study is available in Supplementary Table S5. (**E**) ORA analysis based on the KEGG database for the group of dynamic RBPJ sites identified in Beko cells. Full list of the KEGG terms identified in this study is available in Supplementary Table S5. (**F**) Heat maps showing the effects of a dominant negative mutant of MAML (dnMAML) (68), of GSI washout, apicidin treatment, hypoxia exposure or DMOG treatment on the expression of genes downregulated by GSI and associated with dynamic RBPJ binding sites. Black boxes indicate genes that are not represented in the microarray data from dnMAML-expressing cells. (**G**) Line plot showing the effects of GSI treatment within the gene bodies of those genes significantly downregulated upon GSI and associated with dynamic RBPJ sites on H3K27ac as measured by ChIP-Seq. Outline depciting the standard deviation of the replicates TSS: transcription starting site; TTS: transcription termination site.

In order to investigate the functional consequences of dynamic RBPJ binding we associated the RBPJ sites to nearby genes and we investigated whether gene expression of those genes was influenced by GSI treatment in Beko cells. This was achieved by combining ChIP-Seq data with previously published RNA-Seq data treating Beko cells for 24 h with GSI (16). RBPJ peaks were associated with their target genes using the basal plus extension model of GREAT (42) and effects of GSI treatment on gene expression were analyzed by RNA-Seq: Genes associated with dynamic RBPJ sites were significantly downregulated, while genes associated with static sites were hardly affected (Figure 3A–C and Supplementary Table S3). In support of that, we observed that dynamic sites are statistically more frequently associated with deregulated genes than expected by chance (Supplementary Figure S6A). Genes associated with both dynamic and static RBPJ sites are also enriched for significantly deregulated genes comparable to genes associated with only dynamic RBPJ sites (Supplementary Figure S6A) and we observed a good correlation between changes in gene expression and changes in RBPJ occupancy upon GSI treatment (Supplementary Figure S6B). This indicates that dynamic sites are indeed the drivers for transcriptional response, while static sites seem not to influence the transcriptional response induced by the Notch pathway.

Subsequently, we examined the functions of genes up or downregulated by GSI treatment in Beko cells: Notch-related terms from gene ontology (GO) and KEGG database were identified in the group of genes downregulated by GSI but this was not the case for the upregulated genes (Supplementary Table S4). Furthermore, we examined the functions of genes associated with either static or dynamic RBPJ sites. To this end, we analyzed the latter genes using an overrepresentation analysis (ORA) to test if they were enriched for genes associated with different known biological pathways. The genes associated with dynamic sites were enriched for different Notch-associated terms from GO and KEGG databases (Figure 3D, E and Supplementary Table S5). Furthermore, GO terms associated with negative regulation of development and neurogenesis pathways were significantly enriched within the genes with dynamic RBPJ sites (Supplementary Table S5). The term ‘Th1 and Th2 cell differentiation’ was significantly enriched, when interrogating the KEGG database. This is in line with previous publications, which highlighted the important role of Notch signaling in T helper cell development (57). In contrast, genes associated with static sites were only enriched for the Notch associated KEGG term (‘Notch receptor processing’) based on both GO and KEGG databases (Supplementary Figure S6C, D and Supplementary Table S5). To further validate the findings of the overrepresentation analysis and to correct for gene length mediated biases, identification of significantly enriched pathways was also performed using GREAT (42,43). For genes associated with dynamic sites different GO terms, including ‘Notch signaling pathway’, were enriched (Supplementary Table S6) but this was not the case for static sites (Supplementary Table S6). In addition, the Mouse Phenotype Single KO ontologies identified different T-cell specific phenotypes for genes associated with dynamic sites but not for genes associated with static sites (Supplementary Table S6), in line with the well-known role of Notch signaling in regulating T-cells (58,59). Taken together, dynamic RBPJ sites are strongly correlated with the canonical Notch response including different genes associated with various Notch-mediated functions. We also observed that significantly downregulated genes associated with dynamic RBPJ sites are also downregulated upon overexpression of a dominant negative mutant of the Notch cofactor Mastermind-like (dnMAML) (Figure 3F). We further validated our findings by performing a washout of GSI: We treated Beko cells for 48 h with GSI and after that, we washed out (washout) the inhibitor and placed the cells back in culture for additional 24 h before analyzing their gene expression profile. First of all, we observed that the cleaved and active NICD1 protein reappears in the nuclear fraction upon GSI washout (Supplementary Figure S7A). Overall, the washout experiment matches quite well with our previous GSI gene expression data (16). In fact, genes downregulated by GSI were upregulated upon washout and genes upregulated by GSI are downregulated upon washout (Supplementary Figure S7B–E and Supplementary Table S3). Importantly, genes associated with dynamic RBPJ sites are preferentially upregulated upon GSI washout (Supplementary Figure S7F). Similarly, genes downregulated by GSI and associated with dynamic RBPJ sites are strongly upregulated upon GSI washout (Figure 3F). In addition we reanalyzed our previously published RNA-Seq data upon treatment of Beko cells with apicidin, exposure to hypoxia or treatment with the hypoxia inducer DMOG (16,52): We observed that genes downregulated by GSI and associated with dynamic RBPJ sites are similarly downregulated by these treatments (Figure 3F and Supplementary Table S3).

Having identified genes downregulated upon GSI and associated with dynamic RBPJ sites, we next investigated the effects of GSI treatment on the chromatin state of those genes. We observed reduced levels of H3K27ac, H3K4me3, H3K18ac, H3K9ac and H3K36me3 upon GSI treatment (Figure 3G and S8). However, chromatin accessibility and H3K4me1 are hardly affected at both the TSS and within the gene bodies of genes downregulated by GSI and associated with dynamic RBPJ site(s) (Supplementary Figure S8). In contrast, H3K9me1, H3K9me2, H3K9me3, H3K27me1, H3K27me2, H3K27me3, H3K36me1, H3K36me2 are poorly enriched within gene bodies and not influenced by GSI treatment (Supplementary Figure S8).

Altogether, these data suggest that genes associated with dynamic RBPJ sites are preferentially regulated by the Notch signaling pathway and that the effects of this regulation are reflected in the chromatin dynamics.

### Dynamic RBPJ sites correlate with enhancer size

**Figure 4.**
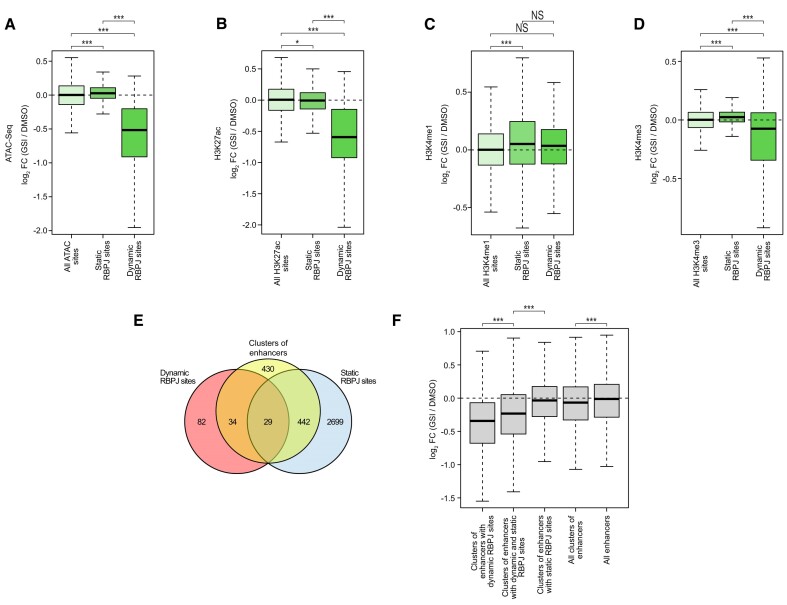
Notch responsiveness on the chromatin is associated with dynamic RBPJ sites. Beko cells were treated for 24 h with 10 μg/ml GSI or DMSO as control. Changes on the chromatin configuration were analyzed by ATAC-Seq and ChIP-Seq (A–D). Box plot showing the effects of GSI treatment on (**A**) chromatin accessibility, (**B**) H3K27ac, (**C**) H3K4me1 and (**D**) H3K4me3 at static and dynamic RBPJ sites as measured by ATAC-Seq and ChIP-Seq. Wilcoxon rank sum tests (*P <0.05, ***P <0.001, NS = not significant). (**E**) Venn diagram showing the overlap of clusters of enhancers with dynamic or static RBPJ sites in Beko cells. (**F**) Boxplot showing the changes in H3K27ac sites within all clusters of enhancers, clusters of enhancers associated with dynamic RBPJ sites, with static RBPJ sites or with both dynamic and static RBPJ sites.

In order to further characterize the Notch transcriptional response, we focused on the analysis of chromatin structure in response to inactivation of Notch signaling at RBPJ sites. We observed reduced chromatin accessibility, H3K27ac, H3K4me3, H3K18ac and H3K9ac while H3K4me1 was unaffected at dynamic RBPJ sites in response to GSI treatment (Figures 4A–D and S9A–B). On the other side, minor but statically significant changes were observed at static RBPJ sites in response to inhibition of Notch signaling: We observed a slight but significant increase in chromatin accessibility, H3K4me1, H3K4me3 and H3K9ac and a slight but significant decrease in H3K27ac and H3K18ac (Figures 4A–D and S9A–B). We also observed a good correlation between changes in RBPJ occupancy and changes in H3K27ac or chromatin accessibility upon GSI treatment in Beko cells (Supplementary Figure S9C, D). Previous works highlighted a dynamic regulation at clusters of enhancers defined as super-enhancers (SEs) in response to Notch signaling (21). In conjunction with the H3K27ac ChIP-Seq data, we used the ROSE tool and detected 935 clusters of enhancers (Supplementary Table S7), which is comparable to the number of clusters of enhancers previously identified in other cells (60). As expected, H3K27ac is strongly enriched over the entire span of the identified clusters of enhancers (Supplementary Figure S10). In addition, we observed that H3K4me1 and H3K18ac and to a lesser extent H3K4me3, H3K9ac and H3K36me1 marks are enriched over the entire length of the identified clusters of enhancers (Supplementary Figure S10). Interestingly, clusters of enhancers are characterized by a mild ATAC-Seq signal indicative of open chromatin (Supplementary Figure S10). Of note, H3K36me2, H3K36me3, H3K27me1, H3K27me2, H3K27me3, H3K9me1, H3K9me2 and H3K9me3 are not enriched at clusters of enhancers (Supplementary Figure S10). Having identified the clusters of enhancers in Beko cells, we proceeded to characterize them in relation to Notch responsiveness. We first observed that a much larger fraction of dynamic RBPJ sites overlap with clusters of enhancers (7.5-fold), compared to static RBPJ sites (3-fold; Figure 4E). Noteworthy, even if a larger proportion of the dynamic sites are in clusters of enhancers, there are still more clusters of enhancers associated with static sites (Figure 4E), which can be explained by the roughly twenty times higher number of static sites. Furthermore, we observed that H3K27ac is preferentially reduced at clusters of enhancers that contain dynamic RBPJ sites compared to the ones that have static RBPJ sites in response to perturbation of Notch signaling (Figure 4F). Together, these data suggest that Notch responsiveness is associated with dynamically regulated RBPJ sites and these dynamic RBPJ sites often are localized within clusters of enhancers.

### Dynamic binding behavior is conserved in triple-negative breast cancer cells

As a next step, we investigated whether distance and binding strength of RBPJ are predictive characteristics of Notch responsiveness also in other cell types. Therefore, we analyzed publicly available datasets from triple negative breast cancer (TNBC) cells (HCC1599 and MB157), in which the Notch signaling pathway has been first blocked with GSI and subsequently reactivated by washout of the inhibitor (19). TNBC is a subtype of breast cancer that is characterized by the lack of ER, PR, HER2 and is known to have active Notch signaling (61). Using RBPJ ChIP-Seq, we identified 14 010 binding sites in HC1599 and 7628 in MB157, respectively (Supplementary Figure S11A,B and S12A-B, respectively). In line with our findings in Beko cells, in both HCC1599 and MB157 cells static and dynamic binding behavior of RBPJ upon washout of GSI was detectable (Supplementary Figures S11A, B and S12A, B, respectively). In HCC1599, 2607 (∼18.6%) of the 14010 RBPJ sites had increased RBPJ binding after washout of GSI (Supplementary Figure S11A, B). In MB157, 2040 of 7628 (∼26.7%) sites were dynamically bound in response to reactivation of Notch signaling (Supplementary Figure S12A, B). Like in Beko cells, the RBPJ binding motif is preferentially enriched at dynamic but not at static sites while the SP binding motif is exclusively enriched at static sites (Supplementary Tables S8 and S9). Having identified dynamic and static RBPJ binding sites in HCC1599 and MB157 cells, we proceeded with their characterization.

In Beko cells, static sites are preferentially enriched at promoter proximal regions, whereas dynamic sites are mostly located at distal regulatory elements (Figures 2A and S2A). In order to test whether this binding behavior is characteristic also of TNBCs, we tested the binding position of dynamic and static RBPJ sites relative to the next TSS in both HCC1599 and MB157. Static RBPJ sites localize much closer to the TSS than the dynamic ones in both cell lines (Supplementary Figures S11C and S12C). We also observed a trend that changes in RBPJ occupancy correlate with changes in gene expression in TNBCs (Supplementary Figures S11D and S12D). Subsequently, we evaluated whether Notch responsiveness on the chromatin level, measured via H3K27ac levels, is preferentially associated with dynamic RBPJ sites. We observed significant changes in H3K27ac preferentially at dynamic RBPJ sites compared to the static ones upon perturbation of Notch signaling (Supplementary Figures S11E and S12E).

**Figure 5.**
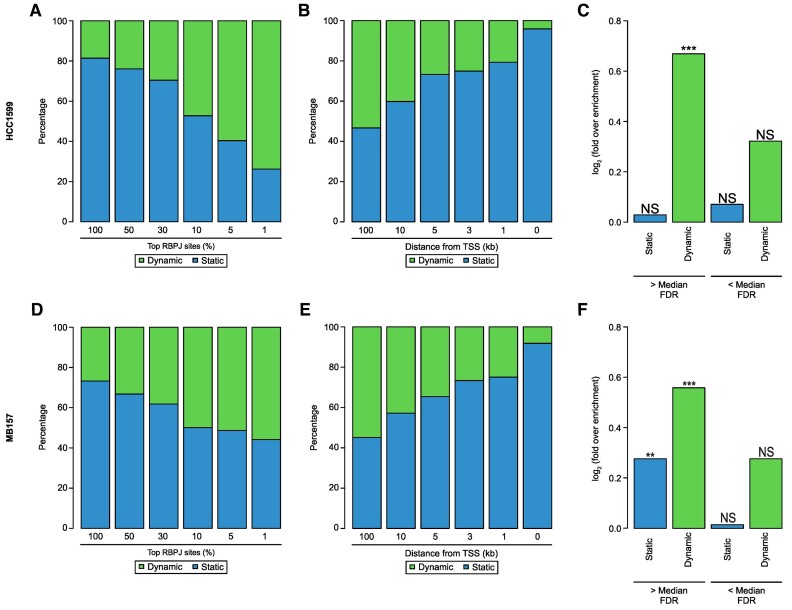
Notch responsiveness is preferentially associated with dynamic RBPJ sites in triple negative breast cancer (TNBC). Publicly available ChIP-Seq and RNA-Seq data were analyzed to investigate Notch responsiveness in HCC1599 (**A–C**) and MB157 (**D–F**) triple negative breast cancer (TNBC) cells. (A and D) Bar plot showing the correlation between static and dynamic RBPJ binding sites and their respective peak quality (MSPC’s p-value) in HCC1599 cells (A) or MB157 cells (D). (B and E) Bar plot showing the correlation between static and dynamic RBPJ binding sites and their distance to the transcription starting site (TSS) in HCC1599 cells (B) or MB157 cells (E). (C and F) Bar plot showing the enrichment of significantly deregulated genes within genes associated with only static or only dynamic RBPJ sites in HCC1599 cells (C) or MB157 cells (F). Additionally, the top half (> Median) and the bottom half (< Median) of all RBPJ sites shown. In these cases, we assigned genes associated with both static and dynamic sites as dynamic. Hypergeometric test (**P <0.01, ***P <0.001, NS = not significant).

Our results in Beko cells suggest that binding strength and positioning of the RBPJ sites are good predictors of Notch responsiveness. Based on these results, we ranked the RBPJ sites in HCC1599 and MB157 by the quality of the peaks as measured by their respective *P*-values. We observed that a high ranking position is strongly correlated with dynamic binding of RBPJ (Figure 5A and D). We observed the inverse trend when looking at the distance to the TSS, where the fraction of dynamic sites becomes smaller with decreasing distance to the TSS (Figure 5B and E). In order to investigate whether there is a connection between Notch-dependent transcriptional response and dynamic sites also in TNBC cells, we tested the statistical enrichment of deregulated genes within the groups of genes that are associated with static or dynamic RBPJ sites. Very similar to Beko cells, we observed a higher enrichment of deregulated genes within the group of dynamic sites (Figure 5C and F).

Taken together, these results suggest that dynamic RBPJ sites are generally located far away from TSSs and are enriched for the high-quality sites in Beko cells and in TNBCs.

### Predicting dynamic and static RBPJ sites

Finally, we used a machine learning algorithm to predict dynamic and static RBPJ sites. This is potentially useful to identify cell type-specific Notch target genes in any given cell-type solely based on RBPJ binding information obtained by ChIP-seq.

**Figure 6.**
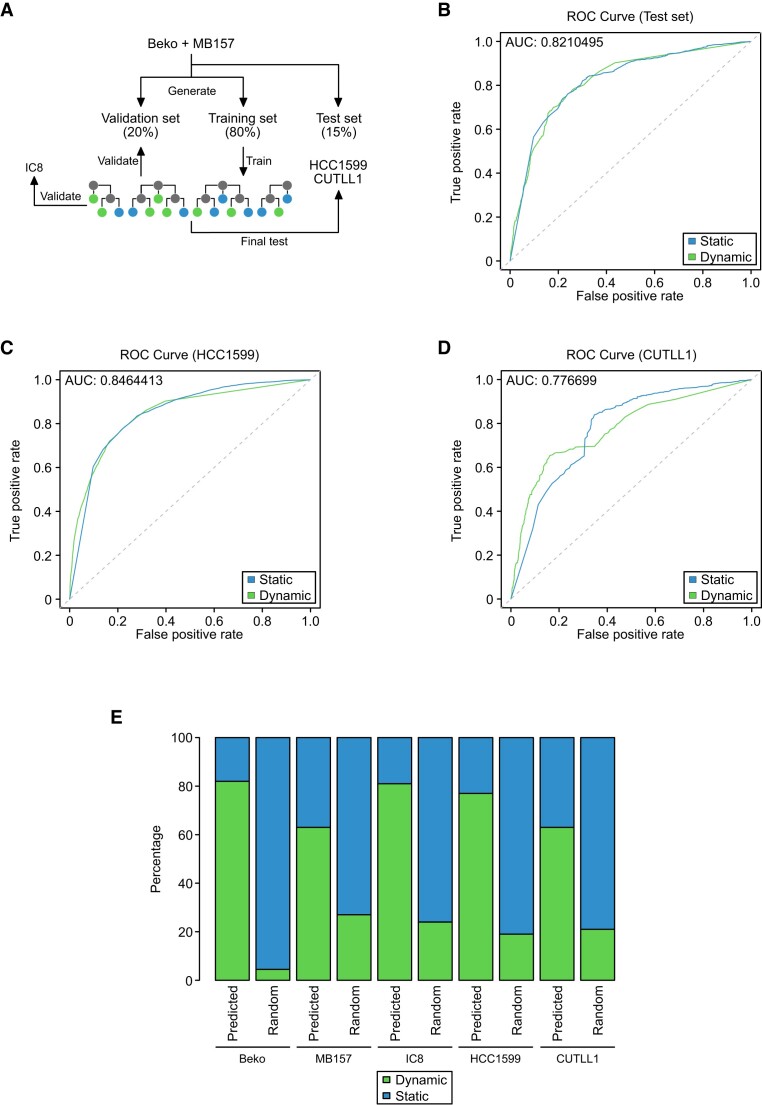
Development of a model for prediction of Notch responsiveness. (**A**) Schematic representation of the approach used to develop and test the prediction model used to identify static and dynamic RBPJ binding sites. (**B**) ROC curves showing the true and false positive rates of the prediction model for static and dynamic sites independently in the test data set (Beko cells + MB157 cells). (**C**, **D**) The prediction model is able to identify static and dynamic RBPJ sites with high accuracy in HCC1599 and CUTLL1. (**E**) Bar plot comparing predicted dynamically bound RBPJ binding sites versus randomly selected RBPJ sites in Beko, HCC1599, MB157, CUTLL1 and IC8 cells as inferred by the random forest approach.

As our training set, we used the combined data from Beko cells and MB157 cells to establish the training set used for the prediction model (Figure 6A). A random forest using the normalized p-value calculated of the peak, the genomic feature (e.g. promoter, gene body, intergenic) and the occurrence of the SP1 and RBPJ motif as a feature for the prediction of static and dynamic RBPJ sites in Beko cells (Figure 6A). The model could efficiently predict both static and dynamic RBPJ sites (Figure 6B, E and Supplementary Table S10). In order to test whether the model is actually able to predict dynamic and static sites in other cell types, we first tested the model on the other TNBC cell line HCC1599. We were able to predict most of the static and dynamic sites in HCC1599 (Figure 6C, E and Supplementary Table S10). Moreover, the 77% of the predicted dynamic sites were true positives.

Finally, we searched for other testable data sets and used data from human T-cell acute lymphoblastic leukemia (T-ALL) called CUTTL1 also employing washout of Notch inhibitor GSI to dynamically regulate the Notch response (21,24,62). We could efficiently identify responsive RBPJ sites in CUTTL1 (Figure 6D, E and Supplementary Table S10). The machine learning approach allows the simple identification of dynamic sites, indicative for Notch responsiveness.

**Figure 7.**
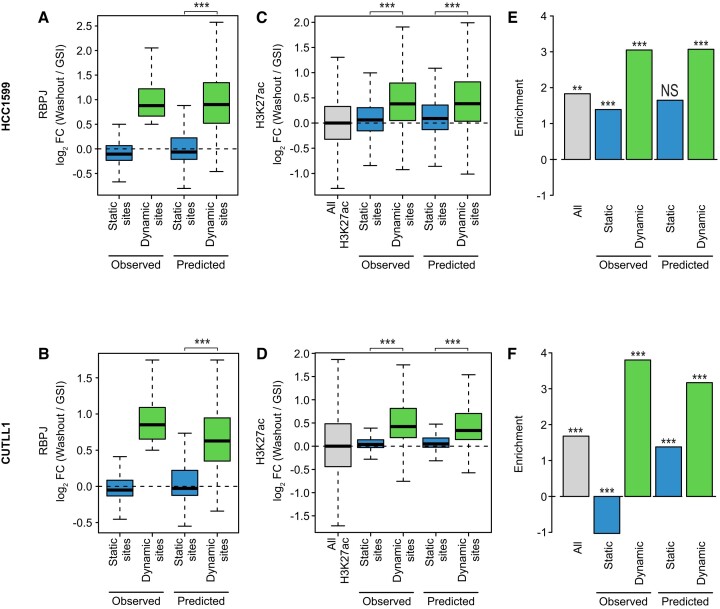
Predicted dynamic sites are comparable to observed ones. (A, B) Box plots showing the RBPJ binding changes upon GSI washout for observed and predicted static or dynamic RBPJ binding sites in HCC1599 (**A**) and CUTLL1 (**B**) cells. (C, D) Box plots showing the H3K27ac changes upon GSI washout for observed and predicted static or dynamic RBPJ binding sites in HCC1599 (**C**) and CUTLL1 (**D**) cells. (E, F) Bar plot showing the enrichment of significantly deregulated genes within genes associated with predicted static or dynamic RBPJ sites in HCC1599 (**E**) and CUTLL1 (**F**) cells. Hypergeometric test (*P <0.05, **P <0.01, ***P <0.001, NS = not significant).

To further validate the efficiency of the predicted dynamic and static sites with the actual measured changes in RBPJ binding upon GSI washout. Of note, the criterion to define an RBPJ site as dynamic was dependent on the changes in Notch activity (log_2_FC > 0.5 for the washout of GSI or < -0.5 for the GSI treatment). In HCC1599 (Figure 7A) and CUTLL1 (Figure 7B) cell lines significant differences between the predicted static and dynamic sites were also detectable. For both cell lines, the predicted dynamic sites had a much stronger increase of RBPJ binding upon washout of GSI compared to the predicted static sites.

An additional feature of dynamic sites was the changes in H3K27ac levels. In order to test, whether these changes were still detectable for the predicted static and dynamic sites, we analyzed H3K27ac ChIP-Seq upon washout of GSI in HCC1599 and CUTLL1. As expected, there is a significant increase in H3K27ac levels at the dynamic versus static sites in both HCC1599 (Figure 7C) and CUTLL1 (Figure 7D). Subsequently, we analyzed the transcriptional response of genes associated with predicted static and dynamic RBPJ sites. The previous analysis showed that genes associated with dynamic sites were more likely significantly differentially regulated in response to Notch signaling. To this end, we used the beforementioned enrichment of differentially expressed genes (DEG) analysis for the groups of predicted static and dynamic RBPJ binding sites. In line with our results in Beko cells, the enrichment of DEGs was much higher for predicted dynamic sites than for predicted static sites for all cell lines under investigation (Figure 7E, F).

In conclusion, the machine learning approach allows to efficiently identify Notch-responsive RBPJ binding sites on a genome-wide scale.

## Discussion

Here, we propose a model for the responsiveness of Notch target genes solely based on the localization, associated motifs and binding strength of the transcription factor RBPJ. We are able to predict functional target sites and thus Notch responsiveness.

When focusing on the differences of sequence context between dynamic and static sites, it is surprising that the dynamic sites carry the canonical RBPJ binding motif ‘TGGGAA’ far more often than the static ones. This could indicate that at dynamic sites RBPJ binds directly to DNA, whereas at static sites the binding may have to be stabilized by additional factors. At the same time these factors may mask certain accessible cofactors or chromatin features and thus prevent activation. We have seen that RBPJ motifs at dynamic and static sites are characterized by subtle differences. Moreover, static RBPJ sites are also marked by additional binding motifs and it remains to be seen whether these contribute to the lack of responsiveness, which may point to a commonly used mechanism. Interestingly, one of top motifs identified at static sites is the RONIN/HCF-1 motif (63,64). This has been described to be specifically associated with housekeeping promoters that are sufficient for robust expression of genes lacking distal enhancers. We speculate that these promoters are particularly robust to perturbations. Noteworthy, the RONIN/HCF-1 motif is composed of two small motifs separated by 2–3 nucleotides, one of which being extremely similar to the RBPJ motif. This might indicate that TFs compete or form a Notch-non-responsive configuration.

In line with this, the vast majority of static sites is associated with TSSs. It has been described that many promoters are resistant to transcriptional perturbations and are tuned to transcriptional robustness. Interestingly, even the shape of the promoter and number of TF binding sites dictate the plasticity of promoters (65,66). Thus, RBPJ binding even together with a coactivating complex might not be decisive in the context of such broad promoters with multiple competing TFs. In contrast to the static sites, a characteristic of a dynamic RBPJ sites could be that the chromatin can possibly form chromatin loops. Apart from looping presence of CTCF sites might also influence the potentially dynamic binding of RBPJ (19,67).

Taken together, it is conceivable that dynamic RBPJ sites represent the well-known Notch targets, while RBPJ at static sites could serve as a ‘gate-keeper’ function and/or be involved in an RBPJ dependent/Notch-independent regulation.

The heterogeneity of the transcriptional outcomes of Notch activation remains a major conundrum. One proposition is that the activation complex consisting of RBPJ/NICD and additional coactivators has enhanced binding activity or that two such coactivator complexes bind cooperatively and this requires two head-to-head RBPJ sites (50,51). Such sites do not account for the majority of dynamic sites determining Notch responsiveness. Our data indicate that enhancer positioning relative to the TSS is more decisive. Our machine learning approach, focusing on RBPJ binding sites and genomic features, not only reduces the time and effort to identify robust Notch responsive genes with good accuracy, but also suggests that this could apply for other TFs factors, such as p53 or TCF of the Wnt signaling pathway. Our data strongly supports the notion that for RBPJ and Notch target genes a locus control region or clusters of enhancers, is important for gene responsiveness. In future, it will be interesting to investigate whether this is also the case for other inducible TFs.

Taken together, computational analyses of TF RBPJ binding combined with distinct genomic features can be used to identify Notch responsiveness in any given cell type. Likely, a comparable model can be established for other inducible systems.

## Supplementary Material

gkae292_Supplemental_Files

## Data Availability

All the data generated in this study have been deposited at GEO under accession number GSE235984. The code to run the prediction of dynamic and static RBPJ sites is available in Zenodo at https://doi.org/10.5281/zenodo.10887370, and at https://github.com/tubcraft/DynamicRBPJ.
