# Supplementary material for: Comprehensive genomic features indicative for Notch responsiveness
Source: Nucleic Acids Res. 2024 Apr 22;52(9):5179–94. doi: 10.1093/nar/gkae292 (PMC11109962; doi:10.1093/nar/gkae292)
Supplement: gkae292_Supplemental_Files [file gkae292_supplemental_files.zip › Suppl_Figures_2003.pdf]

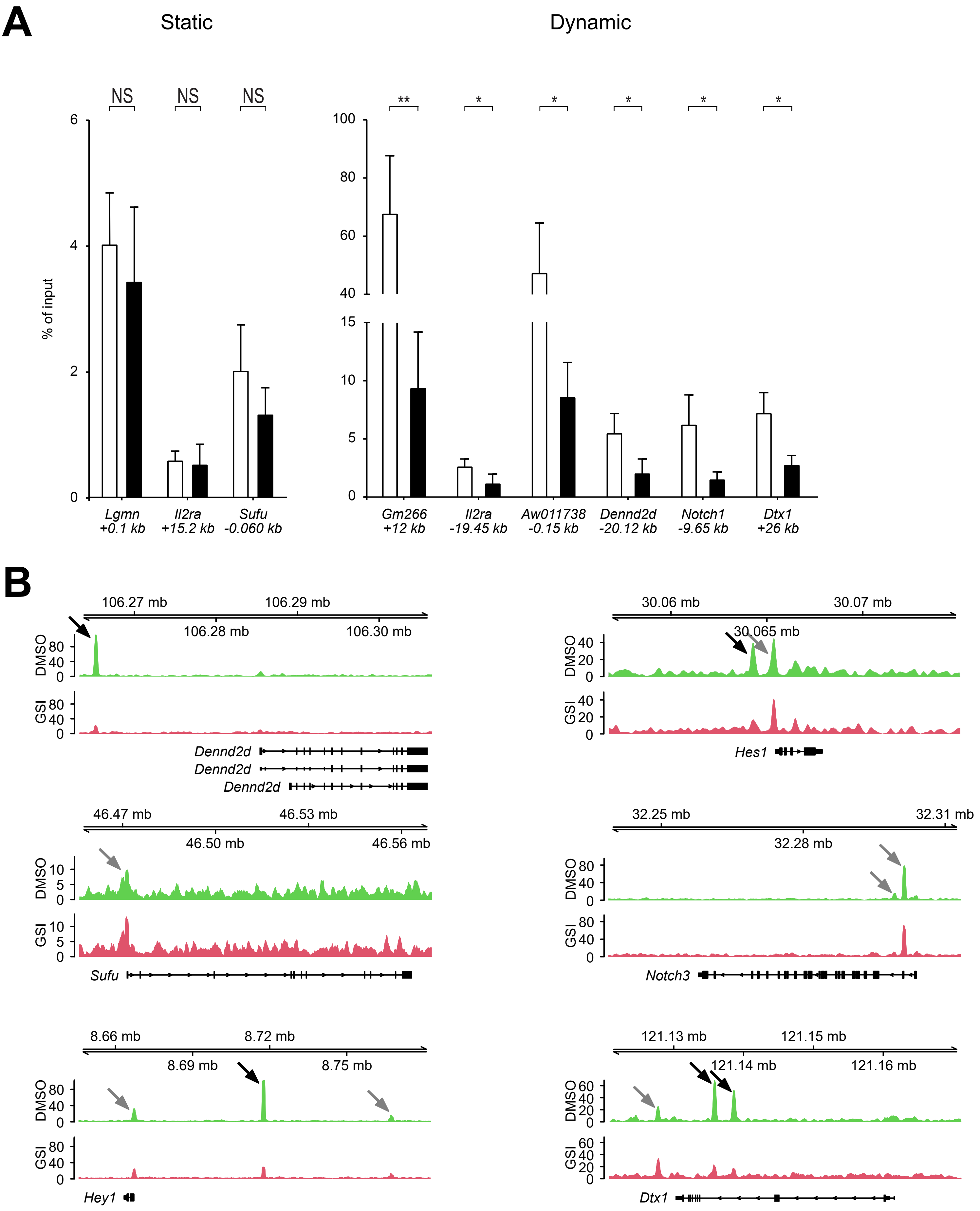

A
Static
Dynamic
NS
NS
NS
**
*
*
*
*
*
% of input
0
2
4
6
0
5
10
15
40
60
80
100
Lgmn +0.1 kb
Il2ra +15.2 kb
Sufu -0.060 kb
Gm266 +12 kb
Il2ra -19.45 kb
Aw011738 -0.15 kb
Dennd2d -20.12 kb
Notch1 -9.65 kb
Dtx1 +26 kb
B
106.27 mb
106.28 mb
106.29 mb
106.30 mb
DMSO
GSI
Dennd2d
Dennd2d
Dennd2d
30.06 mb
30.065 mb
30.07 mb
Hes1
46.47 mb
46.50 mb
46.53 mb
46.56 mb
Sufu
32.25 mb
32.28 mb
32.31 mb
Notch3
8.66 mb
8.69 mb
8.72 mb
8.75 mb
Hey1
121.13 mb
121.14 mb
121.15 mb
121.16 mb
Dtx1

Figure S1

C

Cluster	Motif logos	Motif source	Sites	%	E_value	Most similar motif
Static	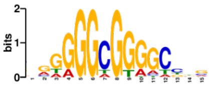	MEME	2247	60.89	1.3e-394	MA0079.3 (SP1)<br>MA0685.1 (SP4)
	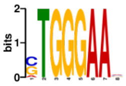	MEME	638	17.29	1.8e-066	MA1116.1 (RBPJ)<br>ZNF75A DBD<br>MA0519.1 (STAT5A::STAT5B)
Dynamic	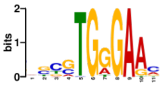	MEME	128	81.01	1.3e-083	MA1116.1 (RBPJ)<br>MA1122.1 (TFDP1)
	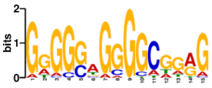	MEME	29	18.35	1.1e-007	MA0079.3 (SP1)<br>MA0516.1 (SP2)<br>MA0599.1 (KLF5)

D

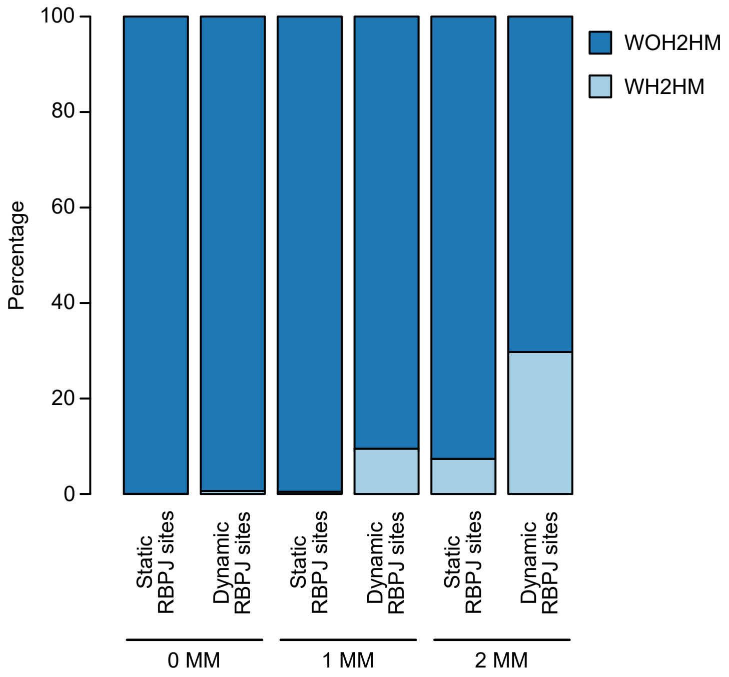

E

Spacer (nt)	Static	Dynamic
15	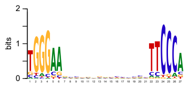	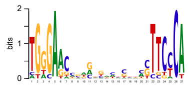
16	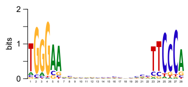	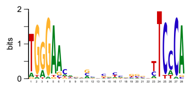
17	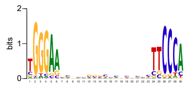	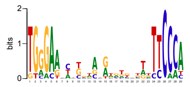

Figure S1_continued

A

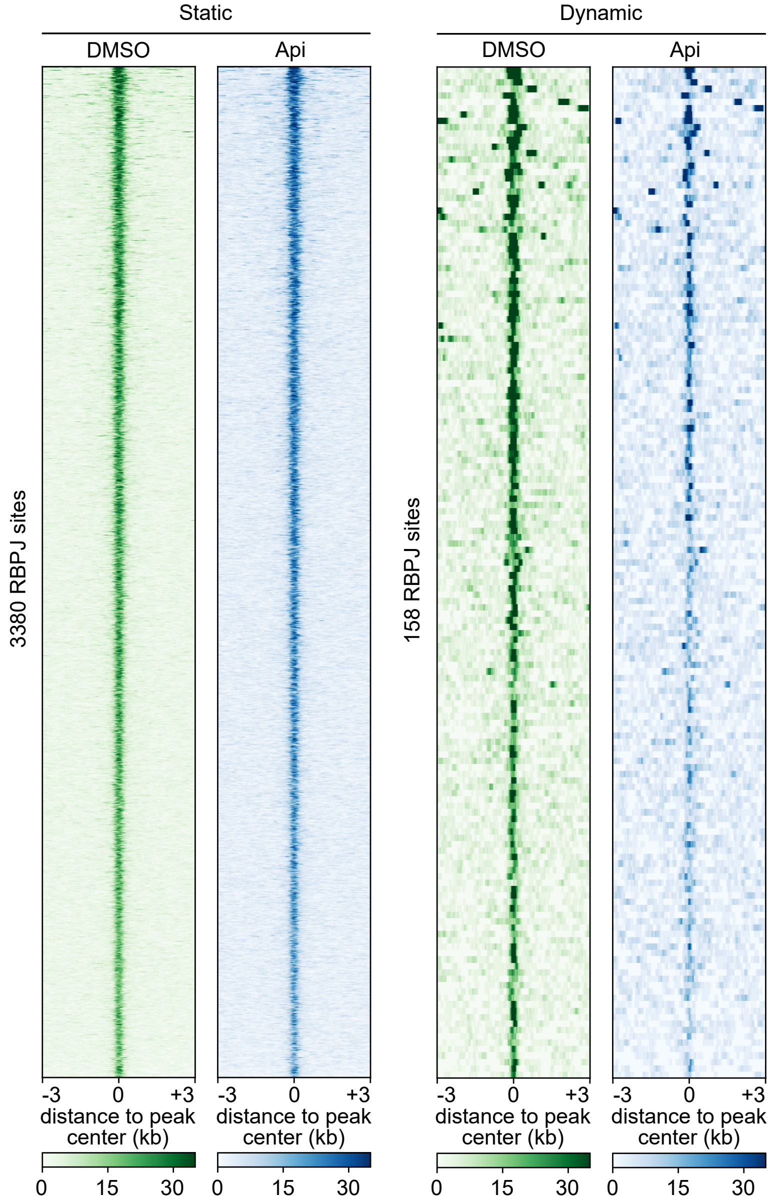

Static
Dynamic
DMSO
Api
DMSO
Api
3380 RBPJ sites
158 RBPJ sites
-3
0
+3
distance to peak center (kb)
0
15
30

B

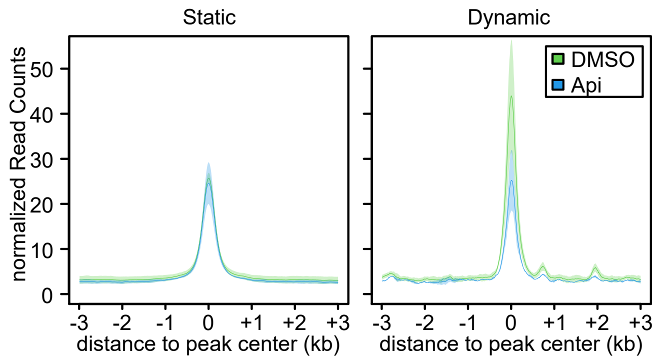

Static
Dynamic
DMSO
Api
normalized Read Counts
0
10
20
30
40
50
-3
-2
-1
0
+1
+2
+3
distance to peak center (kb)

Figure S2

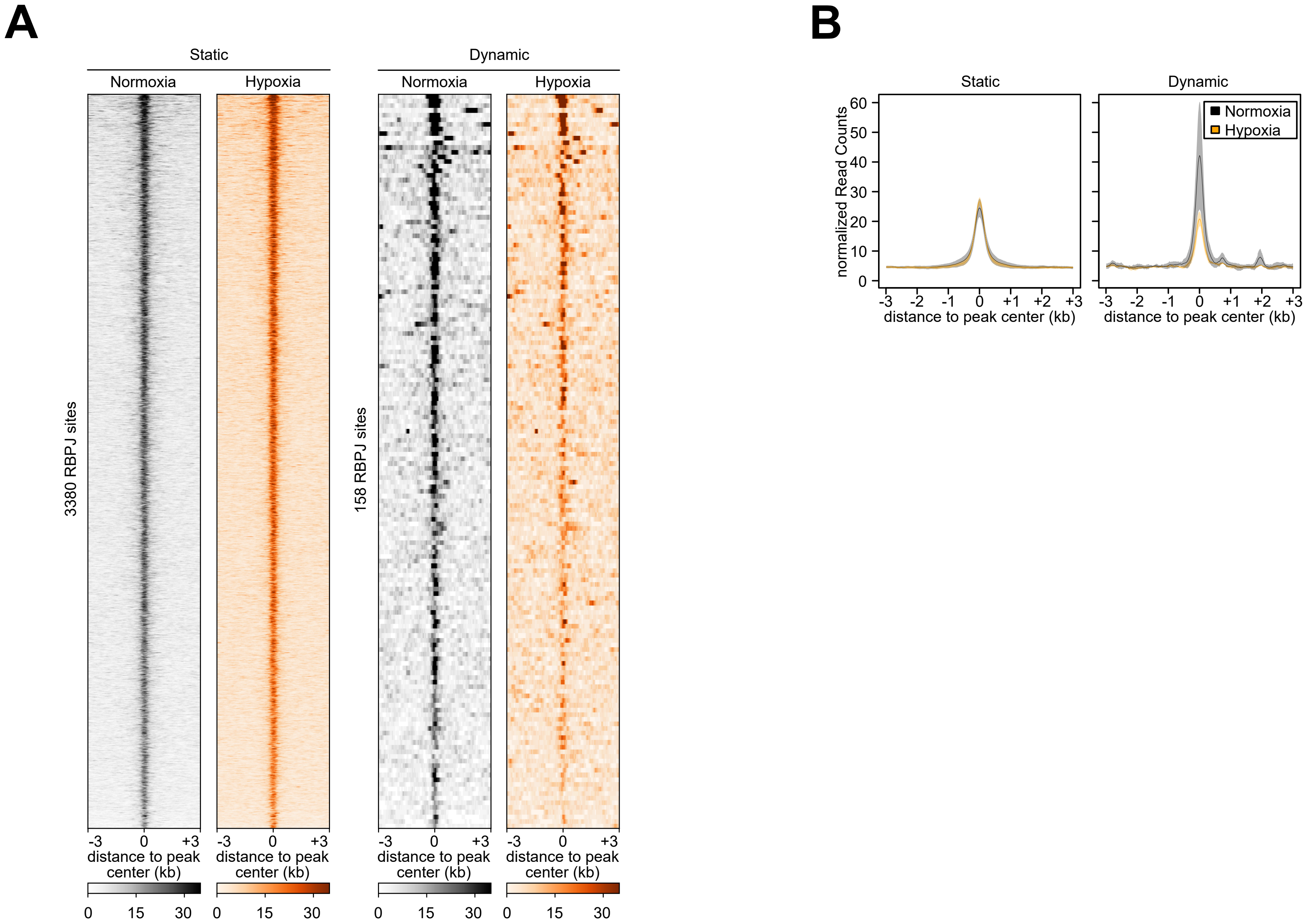
A
Static
Dynamic
Normoxia
Hypoxia
Normoxia
Hypoxia
3380 RBPJ sites
158 RBPJ sites
-3
0
+3
distance to peak center (kb)
0
15
30
B
Static
Dynamic
normalized Read Counts
60
50
40
30
20
10
0
Normoxia
Hypoxia
-3
-2
-1
0
+1
+2
+3
distance to peak center (kb)

Figure S3

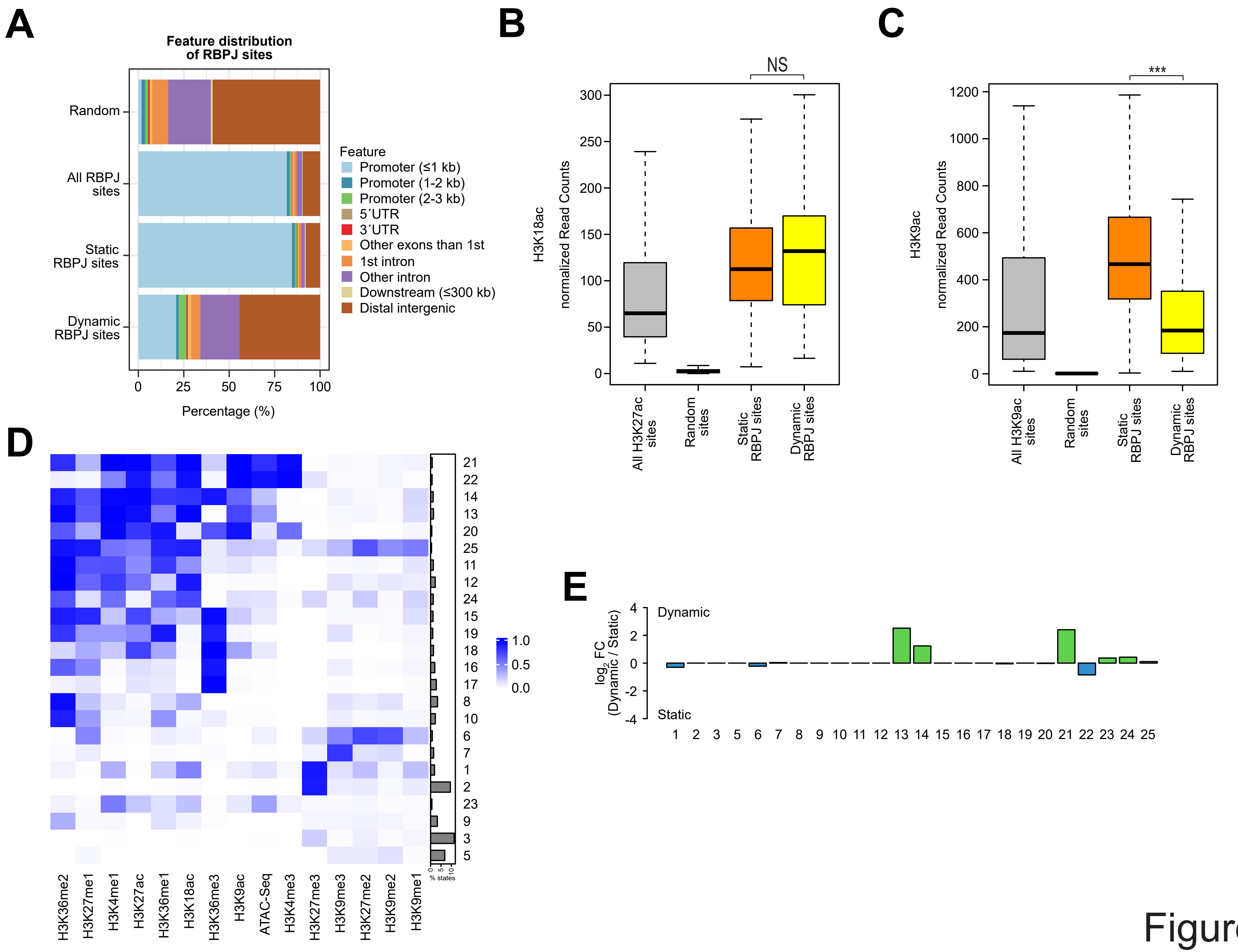
A
Feature distribution of RBPJ sites
Random
All RBPJ sites
Static RBPJ sites
Dynamic RBPJ sites
Feature
Promoter (≤1 kb)
Promoter (1-2 kb)
Promoter (2-3 kb)
5´UTR
3´UTR
Other exons than 1st
1st intron
Other intron
Downstream (≤300 kb)
Distal intergenic
0 25 50 75 100
Percentage (%)
B
NS
H3K18ac
normalized Read Counts
All H3K27ac sites
Random sites
Static RBPJ sites
Dynamic RBPJ sites
C
***
H3K9ac
normalized Read Counts
All H3K9ac sites
Random sites
Static RBPJ sites
Dynamic RBPJ sites
D
H3K36me2
H3K27me1
H3K4me1
H3K27ac
H3K36me1
H3K18ac
H3K36me3
H3K9ac
ATAC-Seq
H3K4me3
H3K27me3
H3K9me3
H3K27me2
H3K9me2
H3K9me1
% states
1.0
0.5
0.0
E
Dynamic
Static
log2 FC (Dynamic / Static)
1 2 3 5 6 7 8 9 10 11 12 13 14 15 16 17 18 19 20 21 22 23 24 25

Figure S4

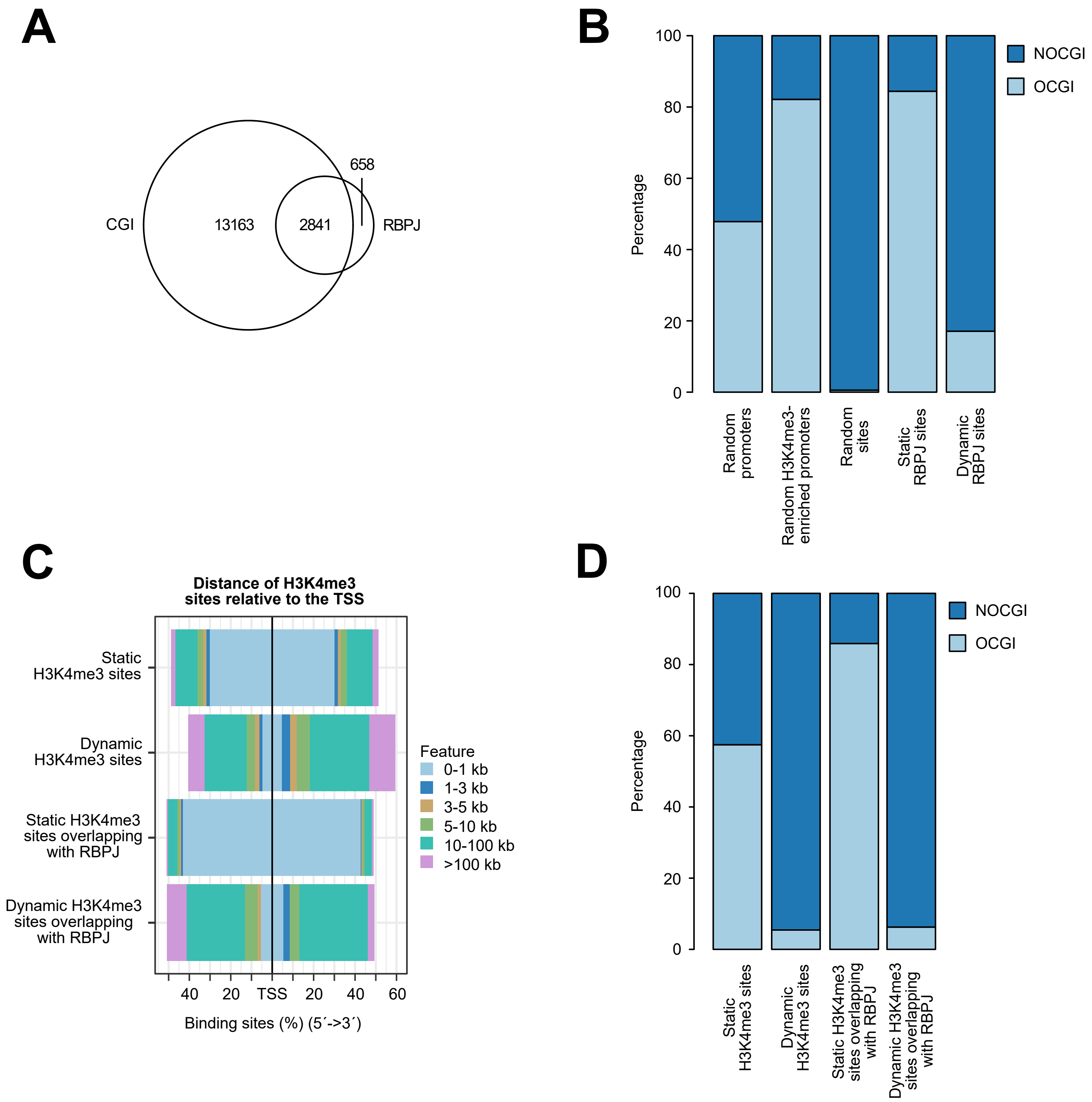
A
CGI
13163
2841
658
RBPJ
B
100
80
60
40
20
0
Percentage
NOCGI
OCGI
Random promoters
Random H3K4me3-enriched promoters
Random sites
Static RBPJ sites
Dynamic RBPJ sites
C
Distance of H3K4me3 sites relative to the TSS
Static H3K4me3 sites
Dynamic H3K4me3 sites
Static H3K4me3 sites overlapping with RBPJ
Dynamic H3K4me3 sites overlapping with RBPJ
40
20
TSS
20
40
60
Binding sites (%) (5´->3´)
Feature
0-1 kb
1-3 kb
3-5 kb
5-10 kb
10-100 kb
>100 kb
D
100
80
60
40
20
0
Percentage
NOCGI
OCGI
Static H3K4me3 sites
Dynamic H3K4me3 sites
Static H3K4me3 sites overlapping with RBPJ
Dynamic H3K4me3 sites overlapping with RBPJ

Figure S5

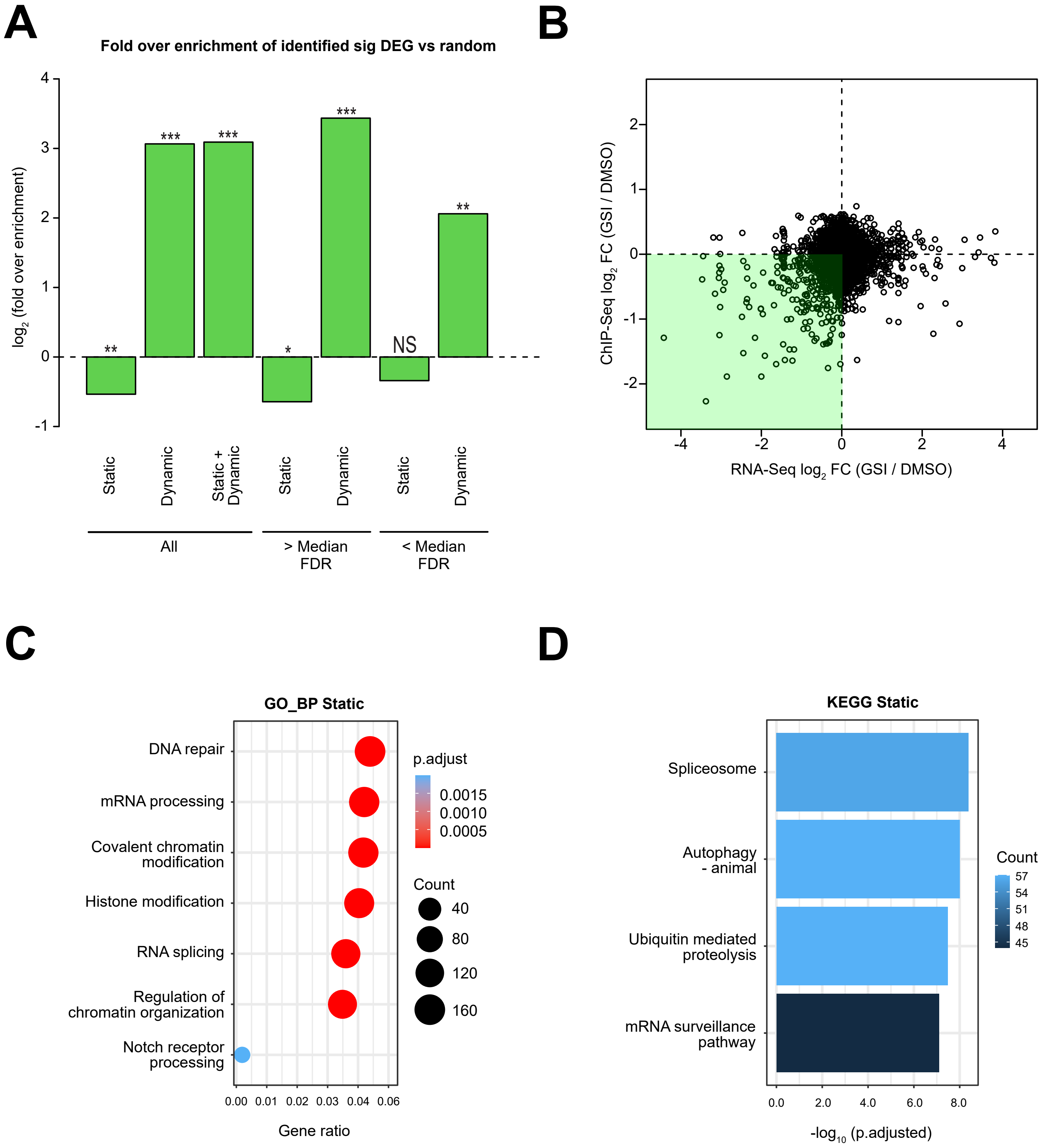
A
Fold over enrichment of identified sig DEG vs random
log2 (fold over enrichment)
4
3
2
1
0
-1
**
***
***
*
***
NS
**
Static
Dynamic
Static + Dynamic
Static
Dynamic
Static
Dynamic
All
> Median FDR
< Median FDR
B
ChIP-Seq log2 FC (GSI / DMSO)
2
1
0
-1
-2
-4
-2
0
2
4
RNA-Seq log2 FC (GSI / DMSO)
C
GO_BP Static
DNA repair
mRNA processing
Covalent chromatin modification
Histone modification
RNA splicing
Regulation of chromatin organization
Notch receptor processing
0.00
0.01
0.02
0.03
0.04
0.06
Gene ratio
p.adjust
0.0015
0.0010
0.0005
Count
40
80
120
160
D
KEGG Static
Spliceosome
Autophagy - animal
Ubiquitin mediated proteolysis
mRNA surveillance pathway
0.0
2.0
4.0
6.0
8.0
-log10 (p.adjusted)
Count
57
54
51
48
45

Figure S6

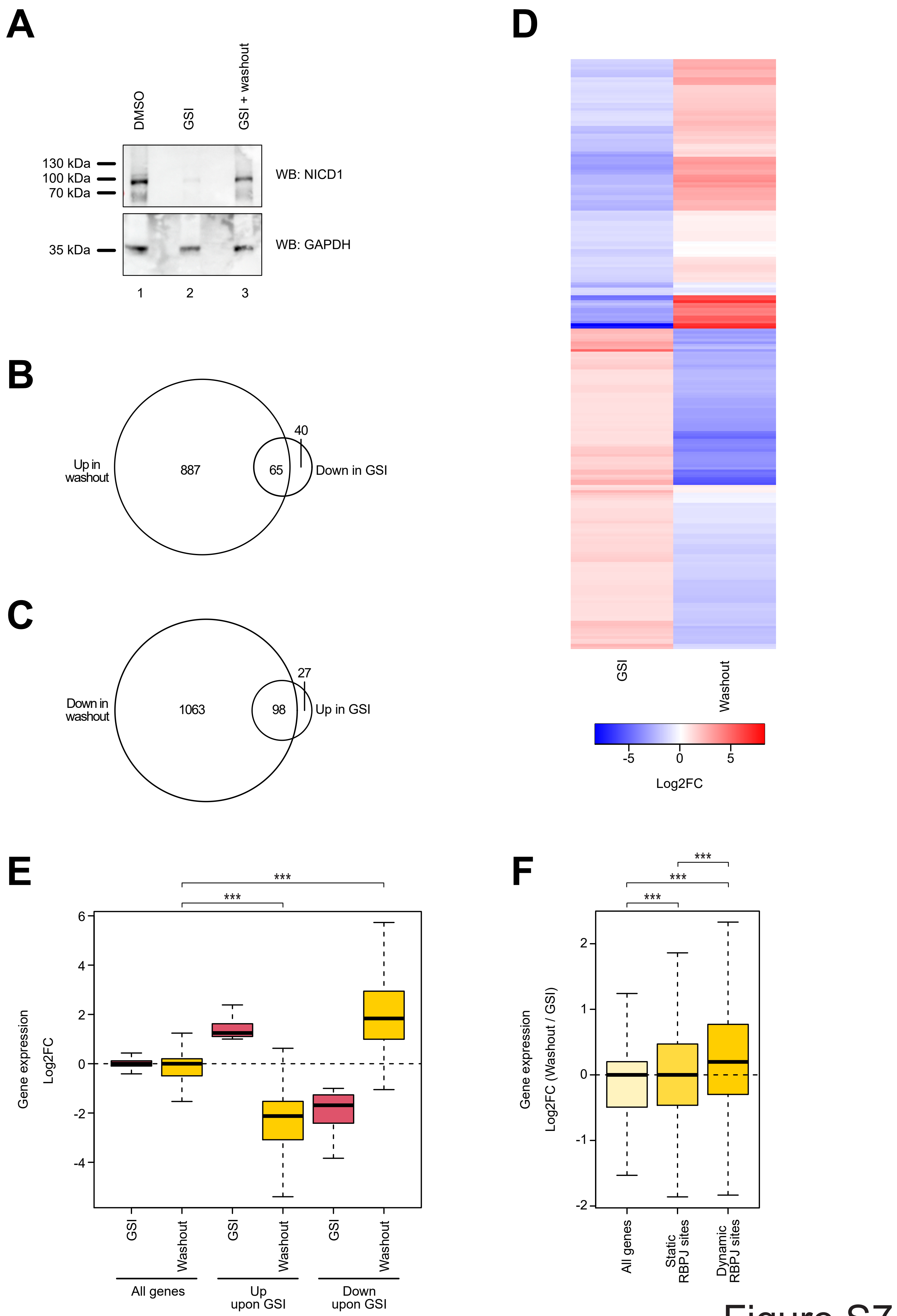

A
DMSO
GSI
GSI + washout
130 kDa
100 kDa
70 kDa
WB: NICD1
35 kDa
WB: GAPDH
1
2
3
B
Up in washout
887
65
40
Down in GSI
C
Down in washout
1063
98
27
Up in GSI
D
GSI
Washout
-5
0
5
Log2FC
E
***
***
Gene expression Log2FC
6
4
2
0
-2
-4
GSI
Washout
GSI
Washout
GSI
Washout
All genes
Up upon GSI
Down upon GSI
F
***
***
***
Gene expression Log2FC (Washout / GSI)
2
1
0
-1
-2
All genes
Static RBPJ sites
Dynamic RBPJ sites

Figure S7

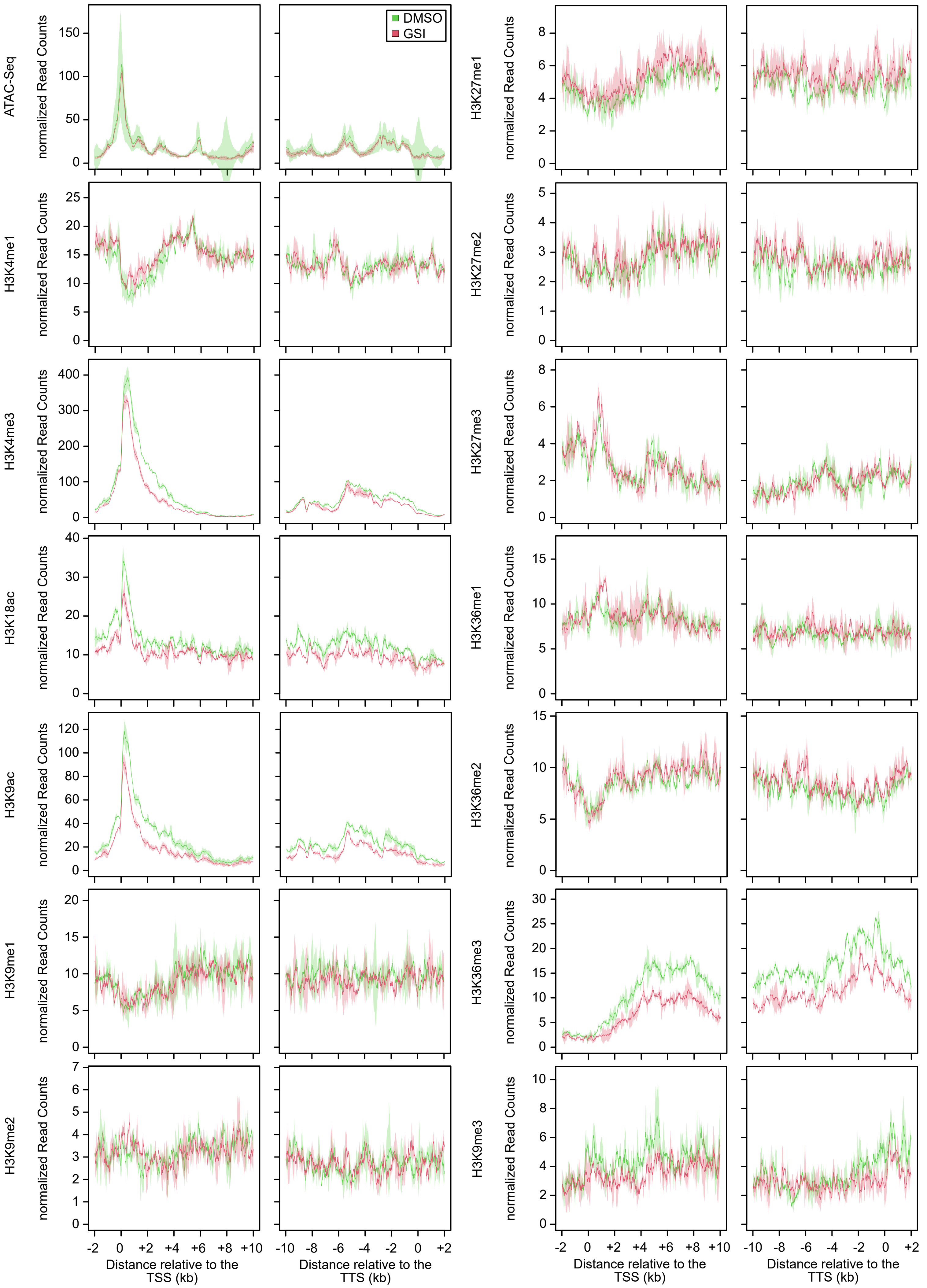
DMSO
GSI
ATAC-Seq
H3K4me1
H3K4me3
H3K18ac
H3K9ac
H3K9me1
H3K9me2
H3K27me1
H3K27me2
H3K27me3
H3K36me1
H3K36me2
H3K36me3
H3K9me3
normalized Read Counts
Distance relative to the TSS (kb)
Distance relative to the TTS (kb)

Figure S8

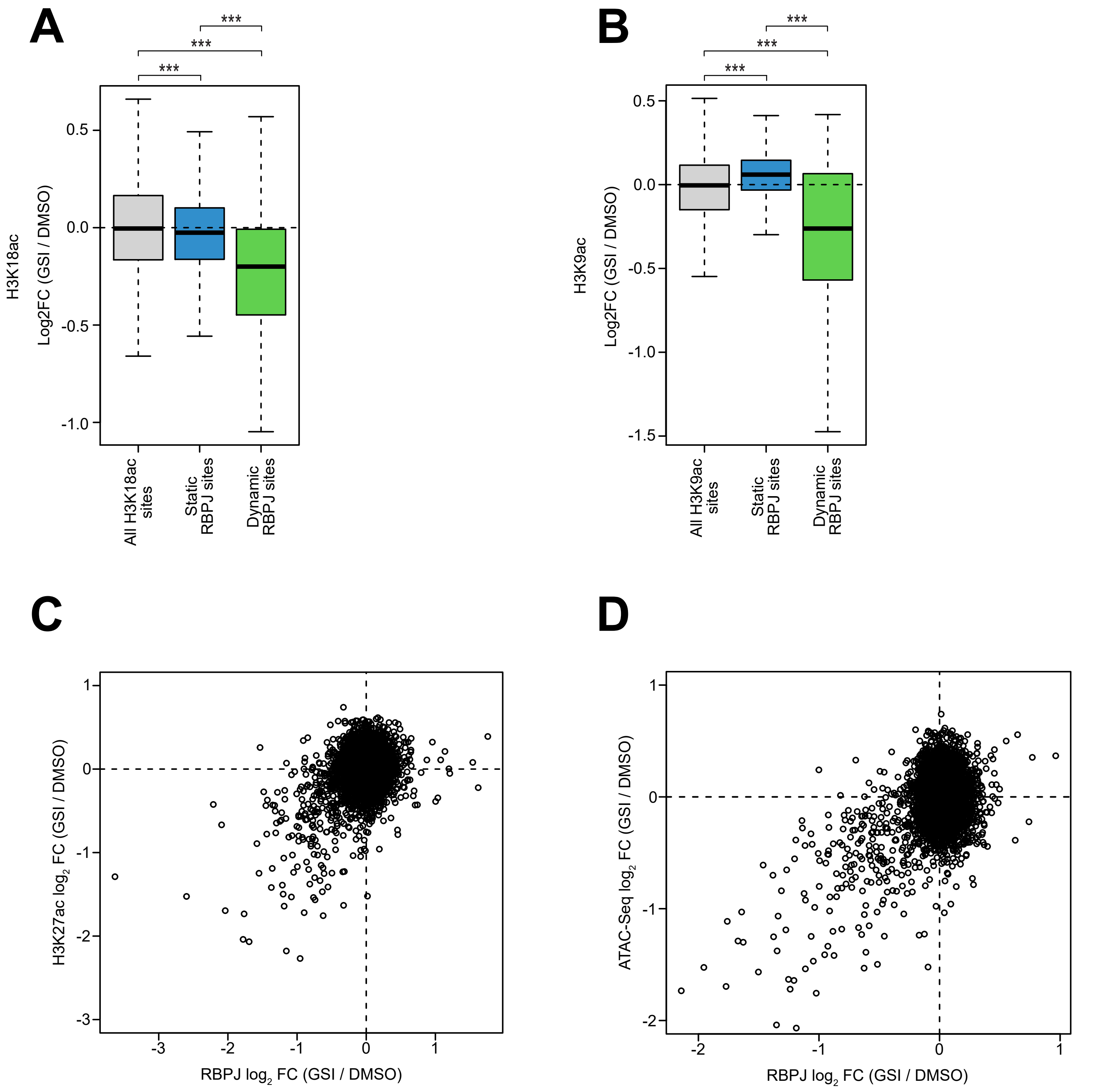
A
B
C
D
***
H3K18ac
Log2FC (GSI / DMSO)
0.5
0.0
-0.5
-1.0
All H3K18ac sites
Static RBPJ sites
Dynamic RBPJ sites
H3K9ac
-1.5
All H3K9ac sites
H3K27ac log2 FC (GSI / DMSO)
RBPJ log2 FC (GSI / DMSO)
ATAC-Seq log2 FC (GSI / DMSO)
1
0
-1
-2
-3

Figure S9

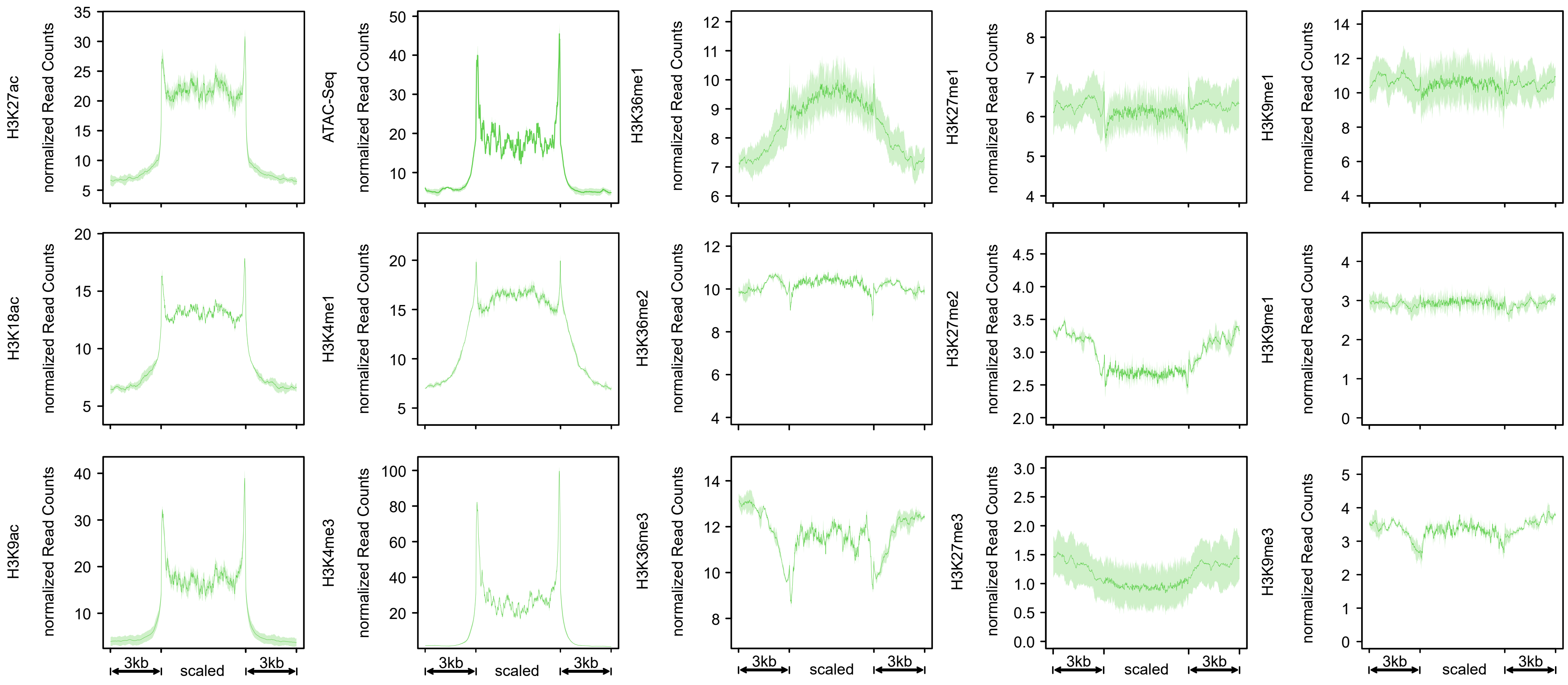
H3K27ac
ATAC-Seq
H3K36me1
H3K27me1
H3K9me1
H3K18ac
H3K4me1
H3K36me2
H3K27me2
H3K9me1
H3K9ac
H3K4me3
H3K36me3
H3K27me3
H3K9me3
normalized Read Counts
3kb
scaled
3kb

Figure S10

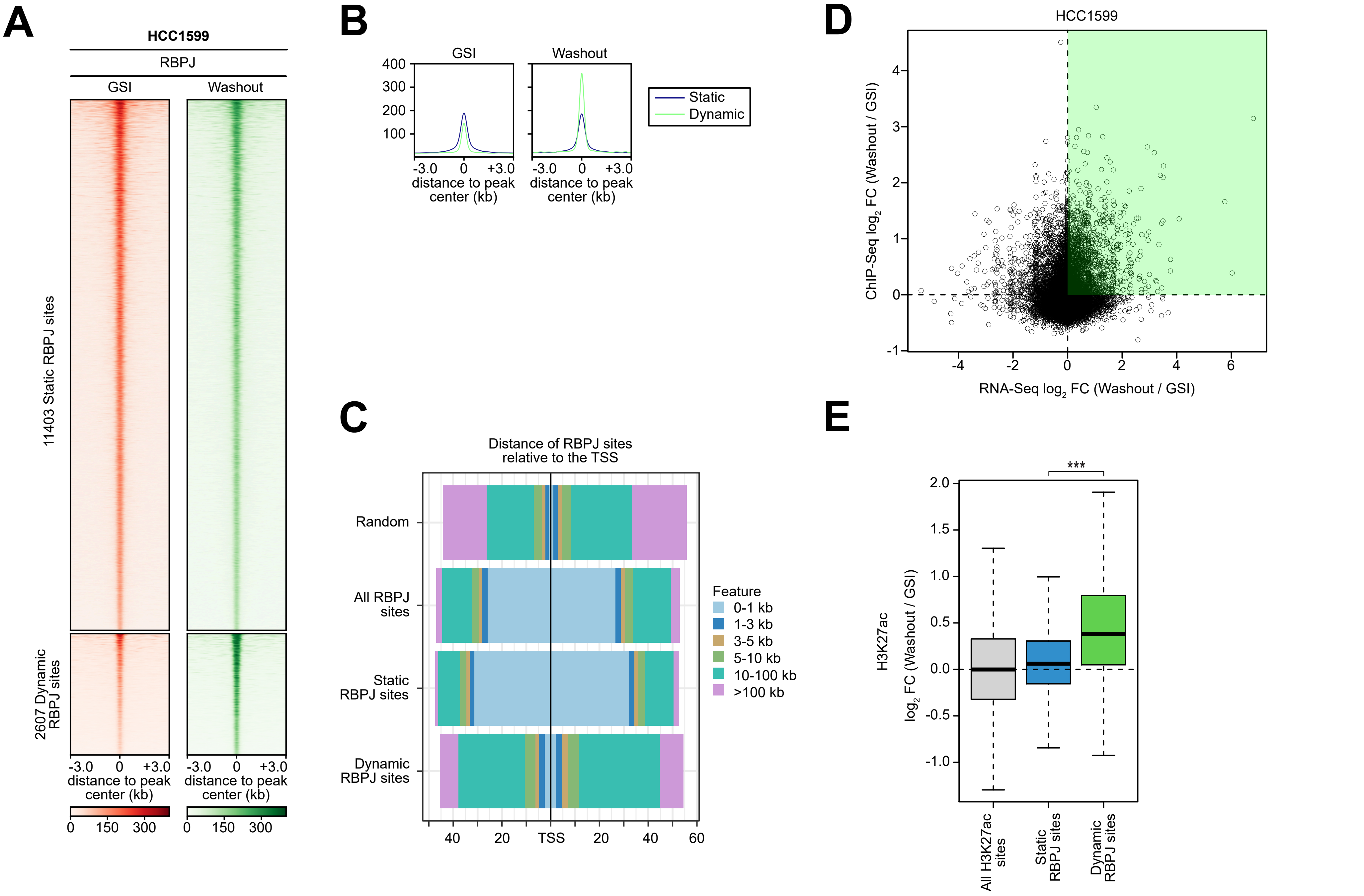
A
HCC1599
RBPJ
GSI
Washout
11403 Static RBPJ sites
2607 Dynamic RBPJ sites
-3.0
0
+3.0
distance to peak center (kb)
0
150
300
B
GSI
Washout
400
300
200
100
Static
Dynamic
distance to peak center (kb)
C
Distance of RBPJ sites relative to the TSS
Random
All RBPJ sites
Static RBPJ sites
Dynamic RBPJ sites
40
20
TSS
20
40
60
Feature
0-1 kb
1-3 kb
3-5 kb
5-10 kb
10-100 kb
>100 kb
D
HCC1599
ChIP-Seq log2 FC (Washout / GSI)
RNA-Seq log2 FC (Washout / GSI)
E
***
H3K27ac
log2 FC (Washout / GSI)
2.0
1.5
1.0
0.5
0.0
-0.5
-1.0
All H3K27ac sites
Static RBPJ sites
Dynamic RBPJ sites

Figure S11

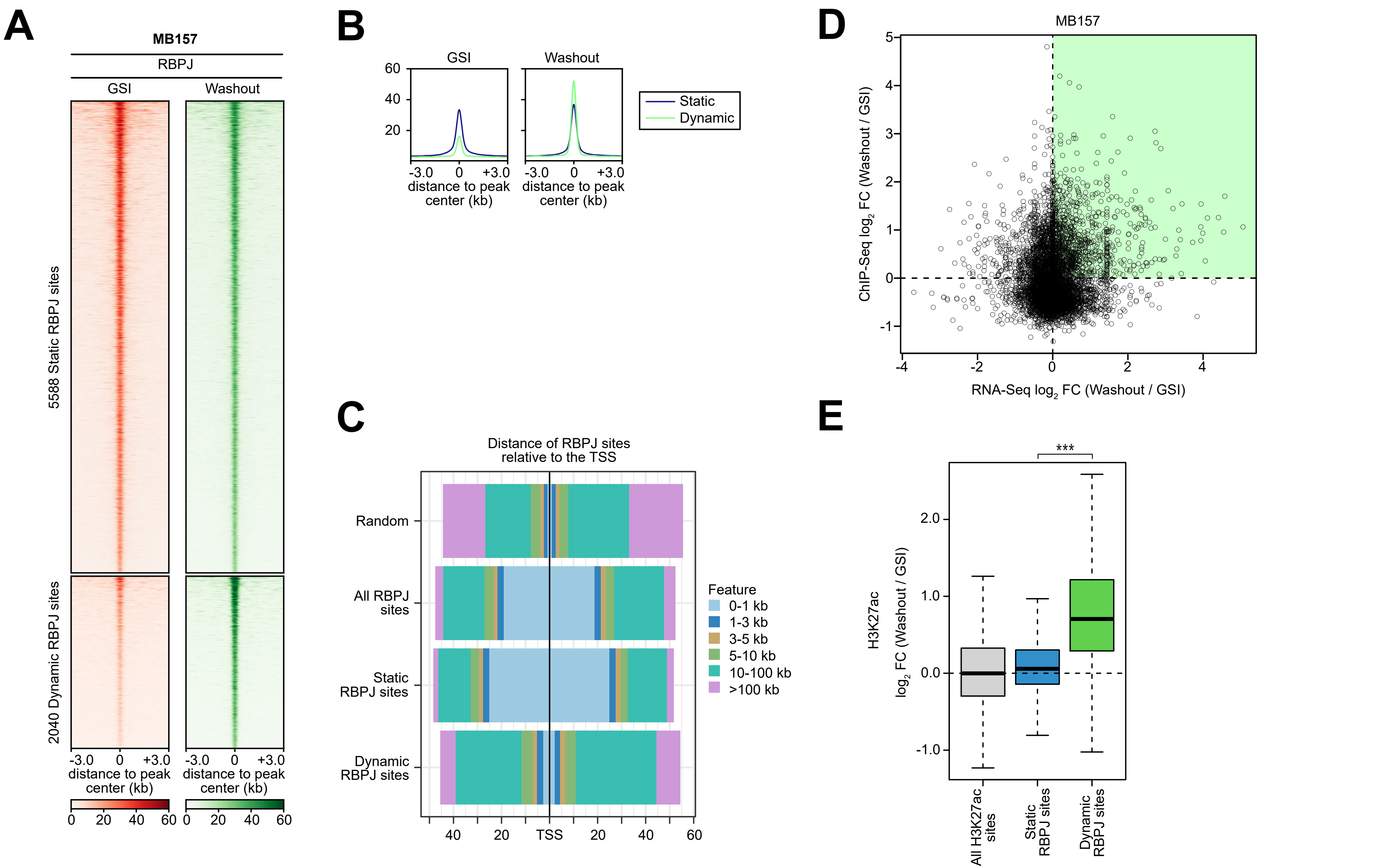
A
MB157
RBPJ
GSI
Washout
5588 Static RBPJ sites
2040 Dynamic RBPJ sites
-3.0
0
+3.0
distance to peak center (kb)
0
20
40
60
B
GSI
Washout
60
40
20
Static
Dynamic
distance to peak center (kb)
C
Distance of RBPJ sites relative to the TSS
Random
All RBPJ sites
Static RBPJ sites
Dynamic RBPJ sites
TSS
Feature
0-1 kb
1-3 kb
3-5 kb
5-10 kb
10-100 kb
>100 kb
D
MB157
ChIP-Seq log2 FC (Washout / GSI)
RNA-Seq log2 FC (Washout / GSI)
E
***
H3K27ac
log2 FC (Washout / GSI)
2.0
1.0
0.0
-1.0
All H3K27ac sites
Static RBPJ sites
Dynamic RBPJ sites

Figure S12
