# Supplementary material for: Comprehensive genomic features indicative for Notch responsiveness
Source: Nucleic Acids Res. 2024 Apr 22;52(9):5179–94. doi: 10.1093/nar/gkae292 (PMC11109962; doi:10.1093/nar/gkae292)
Supplement: gkae292_Supplemental_Files [file gkae292_supplemental_files.zip › z_RBPJ dynamics_supplements_black.pdf]

Comprehensive Genomic Features indicative for Notch Responsiveness

Benedetto Daniele Giaimo[1,^], Tobias Friedrich[1,2,^], Francesca Ferrante[1], Marek Bartkuhn[2,3] and Tilman Borggrefe[1]

[1] Institute of Biochemistry, Justus-Liebig-University Giessen, Friedrichstrasse 24, 35392 Giessen, Germany

[2] Biomedical Informatics and Systems Medicine, Justus-Liebig-University Giessen, Aulweg 128, 35392 Giessen, Germany

[3] Institute for Lung Health, Aulweg 132, 35392 Giessen, Germany

[^]Equal contribution

[*]Corresponding authors:

Tilman.Borggrefe@biochemie.med.uni-giessen.de

or Marek.Barthkuhn@gen.bio.uni-giessen.de

or Benedetto.Giaimo@biochemie.med.uni-giessen.de

Running Title: Specificity of Notch target gene expression is determined by enhancer position and transcription factor binding strength

Files in this data supplement:

Supplemental Figure legends 1, 2, 3, 4, 5, 6, 7, 8, 9, 10, 11 and 12

Supplemental Table legends 1, 2, 3, 4, 5, 6, 7, 8, 9, 10 and 11

Supplemental Table 12

Supplemental figure legends

Figure S1. Identification of static and dynamic RBPJ binding sites in Beko cells. (**A**) Validation of static and dynamic RBPJ sites identified in Beko cells. Beko cells were treated for 24 hours with 10 µg/ml GSI or DMSO as control and the static or dynamic behaviour of selected RBPJ sites was validated by ChIP-qPCR. Shown is the mean ± standard deviation of three independent experiments. (**$P < 0.01$, *$P < 0.05$, NS = not significant, unpaired Student's t-test). (**B**) Representative snapshots showing dynamic RBPJ binding at *Dennd2d*, *Hey1* and *Hes1* and static RBPJ binding at *Sufu*, *Hey1*, *Hes1*, *Notch3* and *Dtx1*. Beko cells were treated for 24 hours with 10 µg/ml GSI or DMSO as control and the static or dynamic binding of RBPJ was investigated by ChIP-Seq. Black arrow(s) indicate dynamic binding site(s) while gray arrow(s) indicate static RBPJ binding site(s). (**C**) Motif discovery analysis of the static and dynamic RBPJ binding sites identified in Beko cells via ChIP-Seq. (**D**) Dynamic but not static RBPJ binding sites are preferentially associated with paired head-to-head RBPJ motifs. Allowing 0-2 mismatches per whole motif. 0 MM: 0 mismatch; 1 MM: 1 mismatch; 2 MM: 2 mismatches. WOH2HM: Without head-to-head RBPJ motifs; H2HM: With head-to-head RBPJ motifs. (**E**) RBPJ binding motifs identified in the group of static and dynamic RBPJ binding sites with a distance of 15, 16 or 17 nucleotides in between the paired head-to-head motifs.

Figure S2. Validation of static and dynamic RBPJ binding sites in Beko cells upon treatment with apicidin. Beko cells were treated for 24 hours with 0.01 µg/ml apicidin or DMSO as control and the genome-wide binding of RBPJ was investigated by ChIP-Seq. (**A**) Heat map showing the static and dynamic RBPJ binding sites identified in control Beko cells and their behavior upon treatment with apicidin (api). (**B**) Line plots showing the average RBPJ binding signal for static and dynamic sites. Outline depicting the standard deviation of the replicates.

Figure S3. Validation of static and dynamic RBPJ binding sites in Beko cells upon exposure to hypoxia. Beko cells were exposed to hypoxia (1% O_2) for 12 hours or kept in normoxia as control and the genome-wide binding of RBPJ was investigated by ChIP-Seq (1). (**A**) Heat map showing the static and dynamic RBPJ binding sites identified in control Beko cells and their behavior upon exposure to hypoxia. (**B**) Line plots showing the average RBPJ binding signal for static and dynamic sites. Outline depicting the standard deviation of the replicates.

Figure S4. Dynamic RBPJ sites represent distal enhancers. (**A**) Genomic features associated with all, static and dynamic RBPJ. The figure includes the genomic background distribution (random). (**B** and **C**) Beko cells were analyzed by ChIP-Seq to characterize the chromatin landscape at static and dynamic RBPJ sites or at random sites and all detected sites of the given mark as control. (**B**) Box plot showing that H3K18ac is similarly enriched at static and dynamic RBPJ sites. (**C**) Box plot showing that H3K9ac is higher at static compared to dynamic RBPJ sites. Wilcoxon rank sum tests (***$P < 0.001$, NS = not significant). (**D**) Heat map showing the enrichment of histone marks and accessibility at the twenty-five generated chromatin states. State 4 was removed, as it corresponds to the absence of any histone marks. (**E**). Bar plot showing the differences of the enrichment of all states at dynamic versus static sites.

Figure S5. (**A**) Venn diagram showing the overlap of RBPJ sites identified in Beko cells with known mouse CpG islands (CGI). (**B**) Static RBPJ binding sites are more often associated with CGI compared to the dynamic ones. NOCGI: Not overlapping with CpG islands; OCGI: Overlapping with CpG islands. (**C**) Distance of static and dynamic H3K4me3 binding sites from the transcription starting site (TSS) of the associated genes. (**D**) Static but not dynamic H3K4me3 sites overlapping with RBPJ binding sites are more often associated with CGI. NOCGI: Not overlapping with CpG islands; OCGI: Overlapping with CpG islands.

Figure S6. Characterization of the Notch-dependent gene expression program in Beko cells. Beko cells were treated for 24 hours with 10 µg/ml GSI or DMSO as control. Effects on gene expression were analyzed by RNA-Seq. (**A**) Bar plot showing the enrichment of significantly deregulated genes within genes associated with only static, only dynamic or dynamic and static RBPJ sites. Additionally, the top half (> Median) and the bottom half (< Median) of all RBPJ sites are shown. Hypergeometric test (**P* < 0.05, ***P* < 0.01, ****P* < 0.001, NS = not significant). (**B**) Scatter plot showing the correlation between changes in RBPJ occupancy and changes in gene expression upon GSI treatment in Beko cells. Highlighted in green are genes downregulated upon GSI treatment associated with RBPJ sites that show reduced binding upon GSI treatment. Pearson correlation coefficient (r): Static RBPJ sites: 0.01616405; Dynamic RBPJ sites: 0.3394885. (**C**) ORA using the gene ontology (GO) database for the group of static RBPJ sites identified in Beko cells. The panels indicate the biological process (BP) analysis. Full list of the GO terms identified in this study is available in Table S5. (**D**) ORA analysis using the KEGG database for the group of static RBPJ sites identified in Beko cells. Full list of the KEGG terms identified in this study is available in Table S5.

Figure S7. Characterization of the effects of Notch induction by GSI washout on gene expression. Beko cells were treated for 48 hours with 10 µg/ml GSI, the GSI was washed out and the cells were placed back in culture for additional 24 hours before performing the experiments. (**A**) The active cleaved NICD1 protein reappears in the nucleus of Beko cells 24 hours after washing out the GSI. Nuclear extracts (NE) were analyzed by Western blotting versus the endogenous cleaved NICD1 protein or H3 as loading control. (**B** and **C**) Venn diagrams showing the overlap between (**B**) genes upregulated (up) upon GSI washout and downregulated (down) upon GSI treatment or between (**C**) genes downregulated (down) upon GSI washout and upregulated (up) upon GSI treatment. (**D**) Heat map depicting genes deregulated upon GSI treatment or GSI washout. (**E**) Box plot showing the quantification of the heat map shown in panel **D** selecting based on genes

upregulated or downregulated upon GSI treatment. (**F**) Box plot showing the effects of GSI washout on the expression of genes associated with static or dynamic RBPJ sites. Wilcoxon rank sum tests (***P < 0.001).

Figure S8. Characterization of Notch responsiveness on the chromatin level in Beko cells. Beko cells were treated for 24 hours with 10 µg/ml GSI or DMSO as control and changes on the chromatin configuration within the gene bodies of those genes significantly downregulated upon GSI and associated with dynamic RBPJ sites were analyzed by ATAC-Seq and ChIP-Seq (H3K4me1, H3K4me3, H3K18ac, H3K9ac, H3K27me1, H3K27me2, H3K27me3, H3K36me1, H3K36me2, H3K36me3, H3K9me1, H3K9me2 and H3K9me3). TSS: Transcription starting site; TTS: Transcription termination site.

Figure S9. Chromatin responsiveness is associated with dynamic RBPJ sites Beko cells were treated for 24 hours with 10 µg/ml GSI or DMSO as control. Changes on the chromatin configuration were analyzed by ChIP-Seq (**A** and **B**). Box plot showing the effects of GSI treatment on (**A**) H3K18ac and (**B**) H3K9ac at static and dynamic RBPJ sites as measured by ChIP-Seq. Wilcoxon rank sum tests (***P < 0.001). (**C**) Scatter plot showing the correlation between changes in RBPJ occupancy and changes in H3K27ac upon GSI treatment in Beko cells. Pearson correlation coefficient (r): Static RBPJ sites: 0.1303332; Dynamic RBPJ sites: 0.4376965. (**D**) Scatter plot showing the correlation between changes in RBPJ occupancy and changes in chromatin accessibility upon GSI treatment in Beko cells. Pearson correlation coefficient (r): Static RBPJ sites: 0.143555; Dynamic RBPJ sites: 0.5735445.

Figure S10. Identification and characterization of clusters of enhancers in Beko cells. Clusters of enhancers were identified in Beko cells based on the H3K27ac signal. Line plots showing the distribution of H3K27ac, H3K18ac, H3K9ac, ATAC-Seq, H3K4me1, H3K4me3, acH3K36me1,

H3K26me2, H3K36me3, H3K27me1, H3K27me2, H3K27me3, H3K9me1, H3K9me2 and H3K9me3 at SEs in Beko cells.

Figure S11. Notch responsiveness is preferentially associated with dynamic RBPJ sites in HCC1599 cells. Publicly available ChIP-Seq data were analyzed to investigate Notch responsiveness in HCC1599 triple negative breast cancer (TNBC) cells. (**A**) The heat map shows the enrichment of RBPJ at static and dynamic RBPJ binding sites in HCC1599 cells. (**B**) Line plot showing the average binding of RBPJ at static and dynamic RBPJ binding sites in HCC1599 cells upon GSI treatment or washout of GSI. (**C**) Distance of all, static and dynamic RBPJ sites to the next transcription starting site (TSS). (**D**) Scatter plot showing the correlation between changes in RBPJ occupancy and changes in gene expression upon GSI washout in HCC1599 cells. Highlighted in green are genes upregulated upon GSI washout associated with RBPJ sites that show increased binding upon GSI washout. Pearson correlation coefficient (r): Static RBPJ sites: -0.01654716; Dynamic RBPJ sites: 0.1613985. (**E**) Box plot showing the effects of GSI washout on H3K27ac at static or dynamic RBPJ sites. Wilcoxon rank sum tests (***$P < 0.001$).

Figure S12. Notch responsiveness is preferentially associated with dynamic RBPJ sites in MB157 cells. Publicly available ChIP-Seq data were analyzed to investigate Notch responsiveness in MB157 triple negative breast cancer (TNBC) cells. (**A**) The heap map shows the enrichment of RBPJ at static and dynamic RBPJ binding sites in MB157 cells. (**B**) Line plot showing the average binding of RBPJ at static and dynamic RBPJ binding sites in MB157 cells upon GSI treatment or washout of GSI. (**C**) Distance of all, static and dynamic RBPJ sites to the next transcription starting site (TSS). (**D**) Scatter plot showing the correlation between changes in RBPJ occupancy and changes in gene expression upon GSI washout in MB157 cells. Highlighted in green are genes upregulated upon GSI washout associated with RBPJ sites that show increased binding upon GSI washout. Pearson correlation coefficient (r): Static RBPJ sites: -0.006016992; Dynamic RBPJ

sites: 0.1067442. (**E**) Box plot showing the effects of GSI washout on H3K27ac at static or dynamic RBPJ sites. Wilcoxon rank sum tests (****P* < 0.001).

Supplementary tables

Table S1. Table showing all identified RBPJ binding sites in Beko cells with their MSPC p-value, binding mode (static or dynamic), DESeq2 results (GSI vs. DMSO), normalized read counts per replicate and the associated gene.

Table S2. MEME-suite results for static and dynamic RBPJ sites in Beko cells.

Table S3. RNA-Seq DESeq2 results and normalized read counts per gene for GSI vs DMSO, Washout vs GSI, dnMAML-ER vs control, apicidin vs DMSO, hypoxia vs normoxia and DMOG vs DMSO.

Table S4. Over-representation analyses based on GO [“Biological Process” (BP)] and KEGG databases for genes down- or upregulated by GSI in Beko cells.

Table S5. Over-representation analyses based on GO [“Biological Process” (BP) and “Molecular functions” (MF)] and KEGG databases for genes associated with static or dynamic sites in Beko cells.

Table S6. Table showing the enrichment of significantly enriched GO terms or Mouse Genome Informatics (MGI) Phenotype ontology for single KO of genes associated with static or dynamic sites using GREAT.

Table S7. Table showing all identified clusters of enhancers in Beko cells with their genomic coordinates.

Table S8. MEME-suite results for static and dynamic RBPJ sites in HCC1599 cells.

Table S9. MEME-suite results for static and dynamic RBPJ sites in MB157 cells.

Table S10. Table showing predicted vs observed RBPJ sites in Beko, MB157, HCC1599, CUTLL1 and IC8 cells.

Table S11. Alignment statics of the data analysed in this study. The table includes the number of reads, number of alignment reads and percentage of alignment. Additionally, GEO numbers and PMIDs are included.

Table S12. Primers used for ChIP-qPCR.

**ChIP**		
	Mus musculus	Probe
Aw011738 -0.15 kb fw	5´-GAT CGG CTA CGC GTA ACA AC-3´	19
Aw011738 -0.15 kb rv	5´-TCC CAG CCA ATG AAG AGC-3	
Dennd2d -20.12 kb fw	5´-AGA CCT CTA CAA AGC ATA GCA ATT T-3	6
Dennd2d -20.12 kb rv	5´-GGG TGT GCA GTG TTG GTC T-3´	
Dtx1 +26 kb fw	5´-GCA TGG GAA CTG TGT TAC AGA A-3´	27
Dtx1 +26 kb rv	5´-CTC TGG GTT GTA GGG GAC AG-3´	
Gm266 +12 kb fw	5´-CCC AGG TGA CTA AGG GAC AC-3´	17
Gm266 +12 kb rv	5´-GAG ACT GAC TGT TCC CAC GAG-3´	
Il2ra -19.45 kb fw	5´-ATT CGG TTG TCA CTG TGG TG-3´	3

Il2ra -19.45 kb rv	5´-TAA CGG TCT AAC CGC CTC AT-3´	
Il2ra +15.2 kb fw	5´-TAC AGC AGT GCC TCC CTT GT-3´	94
Il2ra +15.2 kb rv	5´-CAA CAC TGG ATG TAA CAA CAG AAA-3´	
Lgmn +0.1 kb fw	5´-CTG CAG AAC CGT GGG AAT-3´	83
Lgmn +0.1 kb rv	5´-GCT CAG CGA TCT GTG TTG C-3´	
Notch1 -9.65 kb fw	5´-TCC CTA AGG AGC CAC CAA C-3´	5
Notch1 -9.65 kb rv	5´-TTG CCC ACT TCC TCA ATC C-3´	
Sufu -0.06 kb fw	5´-GGA GGC GGA GTC TAT TGT CA-3´	60
Sufu -0.06 kb rv	5´-TGG CTA GTG TCT TTG CCA GTC-3´	

REFERENCES

1. Ferrante, F., Giaimo, B.D., Friedrich, T., Sugino, T., Mertens, D., Kugler, S., Gahr, B.M., Just, S., Pan, L., Bartkuhn, M. *et al.* (2022) Hydroxylation of the NOTCH1 intracellular domain regulates Notch signaling dynamics. *Cell Death Dis*, **13**, 600.
